# Rational engineering of lung alveolar epithelium

**DOI:** 10.1038/s41536-023-00295-2

**Published:** 2023-04-28

**Authors:** Katherine L. Leiby, Yifan Yuan, Ronald Ng, Micha Sam Brickman Raredon, Taylor S. Adams, Pavlina Baevova, Allison M. Greaney, Karen K. Hirschi, Stuart G. Campbell, Naftali Kaminski, Erica L. Herzog, Laura E. Niklason

**Affiliations:** 1 Department of Biomedical Engineering, Yale University, New Haven, CT USA; 2 Yale School of Medicine, New Haven, CT USA; 3 Department of Anesthesiology, Yale School of Medicine, New Haven, CT USA; 4 Department of Internal Medicine, Section of Pulmonary, Critical Care, and Sleep Medicine, Yale School of Medicine, New Haven, CT USA; 5 Department of Internal Medicine, Yale School of Medicine, New Haven, CT USA; 6 Yale Cardiovascular Research Center, Yale School of Medicine, New Haven, CT USA; 7 Department of Cell Biology, University of Virginia, Charlottesville, VA USA; 8 Cardiovascular Research Center, University of Virginia, Charlottesville, VA USA; 9 Department of Cellular and Molecular Physiology, Yale School of Medicine, New Haven, CT USA

**Keywords:** Regenerative medicine, Tissue engineering, Stem-cell niche, Stem-cell differentiation

## Abstract

Engineered whole lungs may one day expand therapeutic options for patients with end-stage lung disease. However, the feasibility of ex vivo lung regeneration remains limited by the inability to recapitulate mature, functional alveolar epithelium. Here, we modulate multimodal components of the alveolar epithelial type 2 cell (AEC2) niche in decellularized lung scaffolds in order to guide AEC2 behavior for epithelial regeneration. First, endothelial cells coordinate with fibroblasts, in the presence of soluble growth and maturation factors, to promote alveolar scaffold population with surfactant-secreting AEC2s. Subsequent withdrawal of Wnt and FGF agonism synergizes with tidal-magnitude mechanical strain to induce the differentiation of AEC2s to squamous type 1 AECs (AEC1s) in cultured alveoli, in situ. These results outline a rational strategy to engineer an epithelium of AEC2s and AEC1s contained within epithelial-mesenchymal-endothelial alveolar-like units, and highlight the critical interplay amongst cellular, biochemical, and mechanical niche cues within the reconstituting alveolus.

## Introduction

End-stage lung disease remains a leading cause of death in the United States, with more than 150,000 lives lost annually^1^. The only curative treatment, lung transplantation, is plagued by organ shortages and particularly poor graft survival rates^2^. Lung acellular extracellular matrix (ECM) scaffolds provide a pre-made, bioactive template for cellular population and regeneration of functional alveolar networks, which may one day support therapeutic transplantation^3,4^. But despite some reported progress in bioengineered lung revascularization^5,6^, organized epithelial recellularization to reconstitute alveoli with native-like phenotypic features has been an elusive goal^3,4,7–11^. The alveolar epithelium forms a gas-diffusible surface, provides the tightest barrier to fluid leak, and secretes surfactant to prevent alveolar collapse^12^, and as such is indispensable to regenerating functional lung tissue capable of gas exchange.

Alveolar epithelial type 2 cells (AEC2s) are an appealing cell source for alveolar engineering, given their range of critical homeostatic functions^13^ and role as primary epithelial stem cell of the distal lung. In homeostasis and in repair, AEC2s proliferate and differentiate into type 1 AECs (AEC1s), the cells principally responsible for alveolar barrier and for gas exchange^14–16^. Although recent advances have enabled long-term culture of primary^17–19^ or human pluripotent stem cell (hPSC)-derived^20^ AEC2s in vitro, these organoid platforms present a challenge of scalability for acquiring the billions of cells required for human organ culture. Furthermore, accompanying methods of promoting AEC2 differentiation to AEC1s still rely on serum and/or two-dimensional (2D) culture^17–20^, limiting their translatability. Identifying a means to expand, and then differentiate, alveolar epithelium in situ in decellularized lung scaffolds, under defined conditions, would thus represent a significant advance in our efforts to regenerate lung tissue ex vivo.

Prior studies showed that lung ECM alone is insufficient to maintain the differentiated AEC2 phenotype in culture^8,21^ – perhaps an unsurprising finding in light of the numerous and varied signals that comprise the alveolar niche and that act in concert to regulate AEC2 stem cell behavior^22^. Although alveolar fibroblasts (FBs) and their soluble factors have been the primary focus of efforts to dissect the niche^14,23–27^, both endothelial cells (ECs)^28–30^ and immune cells^31,32^ contribute to regulate alveolar regeneration and differentiation. Mechanical tension influences AEC2 differentiation state during early lung development^33,34^ and in regenerating alveoli after pneumonectomy^35^. Yet, there have been few studies applying such niche cues for alveolar engineering. A recent report suggested that soluble AEC2 growth and maturation cues appear to support hPSC-derived AEC2 repopulation of engineered lung; however, lack of comparison to native cells or tissue precludes definitive assessment^7^. Several others have utilized epithelial-endothelial co-culture, but these largely represented proof of concept for repopulating both cellular compartments, and the effects of co-culture remain unclear^3,4,9–11^.

**Fig. 1:**
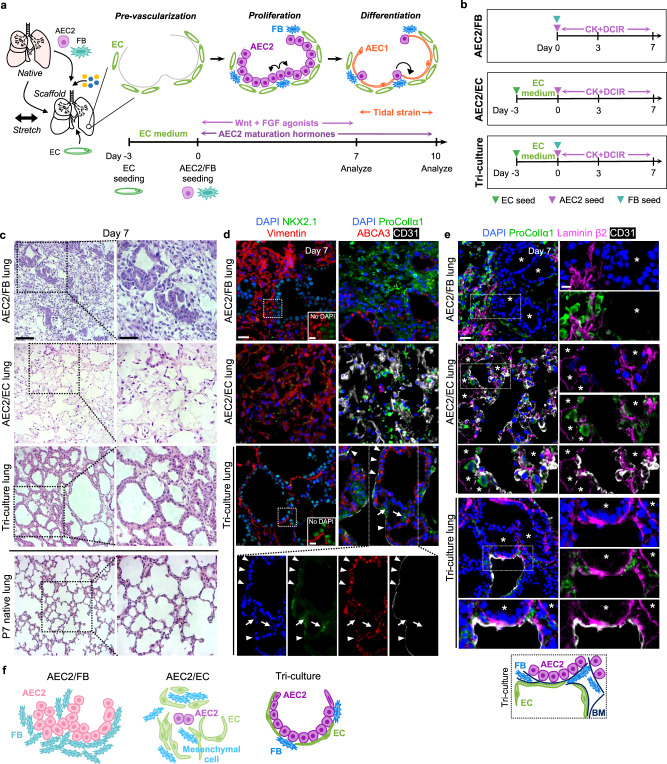
Fibroblast-endothelial co-culture promotes lung scaffold repopulation with AEC2s in organized alveolar-like structures. **a** Schema for alveolar epithelial engineering via modulation of niche cues, with hypothesized timeline of lung recellularization with AEC2s and AEC1s. Timeline corresponds to the pre-vascularization, proliferation, and differentiation phases illustrated at the top of the panel. **b** Timelines for engineered whole lung co-cultures. **c** H&E staining of engineered lung cultures and P7 native lung. Scale bars: main image, 100 μm. Magnified region, 50 μm. **d** Immunostaining showing distribution of NKX2.1^+^ epithelial cells and vimentin^+^ mesenchymal cells (FBs and/or ECs) (left); or ABCA3^+^ AEC2s, ProColIα1^+^ FBs, and CD31^+^ ECs (right) in engineered lungs. Arrowheads, CD31^+^ ECs. Arrows, ProColl1α1^+^ FBs. Scale bar, 25 μm; insets, 10 μm. In images on right, adjustments to contrast of individual color channels were performed identically across all samples. **e** Immunostaining showing localization of ProColIα1^+^ FBs and CD31^+^ ECs relative to basement membrane (BM) component laminin β2 in engineered lungs. Tri-culture cellular and BM organization is schematized below. Asterisks, luminal spaces. Scale bar, 25 μm; insets, 10 μm. **f** Schematics of alveolar repopulation in AEC2/FB, AEC2/EC, and tri-culture lungs at day 7.

Here, we describe the rational engineering of alveolar epithelium in decellularized lung scaffolds, using multimodal niche components as tools to “direct” the AEC2 regenerative process (Fig. 1a). We hypothesized that a phased culture strategy, in which a combination of stromal cells and soluble factors are used to support initial AEC2 expansion, followed by coordination of mechanical strain and select factor withdrawal to drive differentiation of squamous AEC1s in situ, would yield an epithelium of AEC2s and AEC1s, with partial reconstruction of the surrounding alveolus (Fig. 1a). Through systematic investigations in engineered lung, we find that in the presence of an AEC2-specific growth medium, ECs coordinate with FBs to drive repopulation of lung scaffolds with AEC2s that exhibit cardinal molecular and ultrastructural features of native AEC2s, and that secrete surfactant. As revealed by single-cell RNA sequencing (scRNAseq) and in silico ligand-receptor analysis of engineered lung co-cultures, the presence of ECs mitigates the fibrotic milieu observed in the setting of simpler FB co-culture with AEC2s. In the second phase of culture, Wnt and FGF agonist withdrawal synergizes with tidal-level mechanical strain applied to the engineered lung tissue to promote AEC2 differentiation, thereby yielding alveolar-like units containing AEC2s and squamous AEC1s. These results represent a critical advance in the reconstitution of alveolar epithelium, and hence alveoli, for lung cell- and tissue-based regenerative therapies, and provide insights into the diverse niche cues that interact to modulate AEC2 behavior in the alveolus.

## Results

### Fibroblast-endothelial co-culture promotes organized scaffold repopulation with AEC2s

For the initial phase of lung matrix repopulation, we sought to investigate the ability of stromal cell signaling to support AEC2 growth and phenotypic maintenance. Acellular ECM whole rat lung scaffolds^36^ were seeded with AEC2s together with FBs and/or ECs. Primary AEC2s (>70% purity) were isolated from postnatal day (P)7–9 rat pups via magnetic bead-based sorting as previously described^8^ and used immediately for experiments (Supplementary Fig. 1a). The phenotype of isolated AEC2s was confirmed via scRNAseq, with comparison to native P7 AEC2s (Supplementary Fig. 1b–h). Importantly, no contaminating epithelial populations were identified in the AEC2 isolate, including AEC1s (Supplementary Fig. 1b–d, h). Primary neonatal lung FBs were isolated via differential plating from P7–9 rat pups (a timepoint chosen to maximize for lipid-droplet containing lipofibroblasts^37^), and expanded on tissue-culture plastic (TCP) for 72 h prior to seeding in engineered lung scaffolds (Supplementary Fig. 2a–c). scRNAseq of the expanded (passage 1) FB isolate (containing <3% non-mesenchymal cells) revealed subpopulations sharing characteristic markers with each of the mesenchymal populations identified in P7 native lung (Supplementary Fig. 2d–i). However, overall, this starting FB population demonstrated a relatively more pro-migratory and contractile phenotype as compared to native lung mesenchyme (Supplementary Fig. 2j), reflecting the effects of culture on TCP with serum supplementation^38,39^. Primary rat lung microvascular endothelial cells (RLMVECs; from 4–6-week-old rats, VEC Technologies) were expanded to passage 5–6 prior to use (Supplementary Fig. 3a). By scRNAseq, RLMVECs comprise a somewhat heterogenous population of endothelium, which shares some features with general capillary cells, and to a lesser degree with aerocytes and arterial ECs (Supplementary Fig. 3b–j). General capillary-like cells may be a particularly relevant population for engineering purposes, given their role as a capillary stem/progenitor cell^40^.

We seeded AEC2s together with FBs via the airway of decellularized lung scaffolds. For endothelium-containing cultures, ECs were seeded via the pulmonary artery and veins^41^ 3 days prior to the addition of AEC2s and FBs via the trachea (day 0), and cultured in EC-supportive, serum-containing medium to promote “pre-vascularization” of the lung scaffolds (Fig. 1a, b). A serum-free, chemically-defined AEC2-supportive culture medium was utilized from day 0 of culture, containing CK + DCI additives (CHIR99021 [CHIR], keratinocyte growth factor [KGF], dexamethasone, cyclic AMP [cAMP], and 3-isobutyl-1-methylxanthine [IBMX]) that have previously been shown to support AEC2 proliferation and/or maturation^20,42^. This medium was supplemented with retinoic acid to further support epithelial differentiation (final medium dubbed “CK + DCIR” medium) (Fig. 1b). The intact whole lungs were then cultured in bioreactors under pulsatile vascular perfusion for 7 days^43^.

Consistent with the known AEC2 niche-supportive role of FBs in vitro and in vivo^14,25,27^, AEC2/FB co-culture promoted epithelial expansion and repopulation of lung scaffolds with numerous NKX2.1^+^ and ABCA3^+^ AEC2s after 7 days (Fig. 1c, d and Supplementary Fig. 4a–f). However, AEC2/FB lungs comprised disorganized rings and clusters of epithelia within a substantial interstitium of FBs and ECM (Fig. 1c–e and Supplementary Fig. 4a). Interestingly, immunostaining for laminin β2 demonstrated that the epithelial structures in AEC2/FB lungs were nearly devoid of an underlying basement membrane (Fig. 1e).

In contrast, when AEC2s were cultured in lung matrices with ECs but no FBs (“AEC2/EC”), AEC2-like cells were sparse after 7 days, despite initial AEC2 engraftment not significantly different from that in AEC2/FB lungs (Fig. 1c, d, Supplementary Fig. 4a–d, f, and Supplementary Fig. 5a). Abundant ECs were identified in AEC2/EC lungs, along with scattered procollagen Iα1^+^ and alpha smooth muscle actin (αSMA)^+^ FB-like cells (Fig. 1d, e and Supplementary Fig. 5b, c). In a separate experiment, we determined that these FB-like cells were derived from cells of the primary AEC2 isolate (Supplementary Fig. 5d, e), and we speculate that they most likely represent outgrowth of the rare mesenchymal contaminant in the original cell population (Supplementary Fig. 1b, c). The failure of AEC2 growth in AEC2/EC lungs shows the importance of robust numbers of FBs to support initial AEC2 growth and maintenance in this engineered lung model. Thus, in native lung matrix and in the setting of an AEC2 growth medium, FB-only co-culture supports AEC2 growth but yields a tissue structure incompatible with gas exchange, while EC-only co-culture fails to support AEC2 growth and/or phenotypic maintenance (Fig. 1f).

Strikingly, scaffolds that were seeded with all three lineages – FBs, ECs, and AEC2s (“tri-culture”) – exhibited abundant, organized rings of cuboidal AEC2s by day 7, and a tissue structure much more similar to that of neonatal lung (Fig. 1c, d and Supplementary Fig. 4a–f). The epithelial rings were surrounded in most regions by a laminin β2-containing basement membrane (Fig. 1e). CD31^+^ ECs were often located basolateral to the cuboidal AEC2 monolayers, beneath a basement membrane, suggesting close interaction between the endothelium and regenerating epithelium (Fig. 1d, e). Compared to AEC2/FB co-cultures, procollagen Iα1^+^ FBs were relatively scarce. Importantly, despite being seeded into the airway, FBs appeared to be separated from both the AEC2s and the ECs by basement membrane, as in the native lung (Fig. 1e)^44^. This observation is consistent with a prior report showing that FBs can migrate into the interstitium of decellularized lung matrices^45^. Together, these data support the essential role for FBs within the engineered AEC2 niche, but suggest that ECs may additionally contribute to 3D alveolar organization in a manner that is dependent on FB co-culture – yielding AEC2 epithelium with improved localization relative to other cell types (Fig. 1f). Importantly, neither CK + DCIR medium alone, nor endogenous tri-lineage signaling (i.e. without CK + DCIR additive supplementation), supported organized lung scaffold repopulation with AEC2s (Supplementary Fig. 6).

### Endothelial co-culture enhances AEC2 phenotype in the presence of fibroblasts

**Fig. 2:**
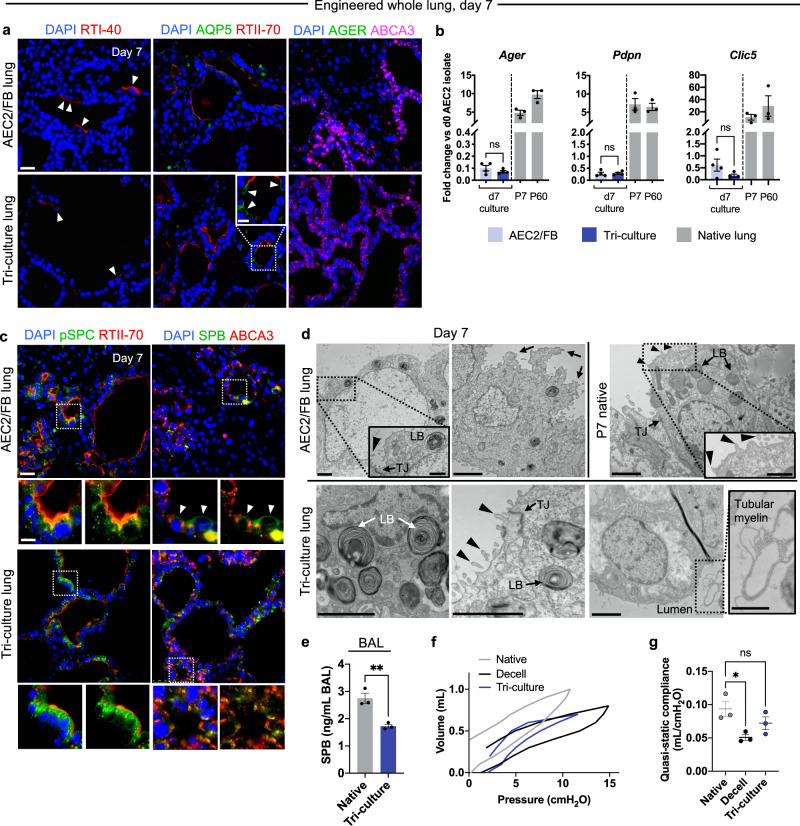
Endothelial co-culture enhances AEC2 phenotype in the setting of fibroblasts. **a** Immunostaining for AEC1 markers RTI-40, AQP5, and AGER in d7 engineered lungs. Arrowheads, cells staining positive for AEC1 markers. **b** qRT-PCR of AEC1 gene expression in d7 engineered lungs; *n* = 4 lungs. Gene expression in native P7 and P60 rat lungs is normalized to the average expression in day 0 AEC2 isolates and shown for approximate comparison only; *n* = 3 lungs. **c** Immunostaining of AEC2 proteins in AEC2/FB and tri-culture lungs. Arrowheads indicate diffuse cytoplasmic SPB labeling in AEC2/FB co-cultured epithelium. **d** TEM examining epithelial ultrastructural features in engineered lungs, with a P7 native AEC2 shown for comparison. LB lamellar body, TJ tight junction. Arrowheads, apical microvilli. Arrows, irregular apical projections. Scale bars, 2 μm. Magnified regions, 1 μm. **e** Quantification of secreted SPB protein in bronchoalveolar lavage (BAL) of adult rat (“Native”) and tri-culture engineered lungs; *n* = 3 lungs. Representative quasi-static pressure-volume (PV) curves (**f**) with calculation of compliance (**g**) for native, decellularized, and tri-culture lungs; *n* = 3 biological replicates. Scale bars in immunofluorescence images, 25 μm. Magnified regions, 10 μm. Error bars indicate the mean ± SEM. **b**, **e** Unpaired two-tailed *t*-test. **g** One-way ANOVA with Holm–Sidak’s multiple comparisons tests. ns not significant, *P < 0.05, **P < 0.01.

Because ECs alone did not appear to support AEC2 growth or phenotypic maintenance in engineered lungs, subsequent analyses focused on AEC2/FB and tri-culture lungs. Notably, examination for canonical AEC1 marker expression in both AEC2/FB and tri-culture lungs by immunostaining (Fig. 2a) and by qRT-PCR of whole lung tissue (Fig. 2b) suggested only rare differentiation of AEC2s into AEC1s in either setting. However, despite similar whole-tissue AEC2 gene expression in AEC2/FB and tri-culture lungs (Supplementary Fig. 4f), close inspection revealed important differences in AEC2 phenotype between the two conditions. By immunostaining, tri-culture AEC2s expressed punctate pro-surfactant protein (SP)C and co-localized SPB/ABCA3, consistent with protein localization to lamellar bodies (Fig. 2c). Transmission electron microscopy (TEM) confirmed the presence of well-formed lamellar bodies in tri-culture AEC2s, as well as characteristic apical microvilli and tight junctions (Fig. 2d). Tri-culture AEC2s were also associated with luminal tubular myelin, a structure characteristic of secreted surfactant (Fig. 2d)^46^. In contrast, AEC2/FB lung AEC2s demonstrated only weak surfactant protein expression, without consistent punctate localization by immunostaining (Fig. 2c). By TEM, co-culture AEC2s exhibited aberrant epithelial cell ultrastructure, including enlarged nuclei and irregular apical projections (Fig. 2d). These features suggest a loss of the appropriate AEC2 cellular program in the setting of FB-only co-culture in lung scaffolds.

To assess functional secretion of surfactant protein within tri-culture engineered lungs, we quantified SPB in epithelial lining fluid obtained via bronchoalveolar lavage (BAL). In day 7 tri-culture engineered lungs, the BAL fluid contained SPB exceeding 60% of native adult rat lung BAL levels, a quantity that may be sufficient to confer physiologic surfactant function in vivo (Fig. 2e)^47^. To assess whole lung mechanical compliance, we generated pressure-volume curves. While decellularized scaffolds had a compliance that was significantly less than that of native lung, the compliance of engineered lungs upon initial expansion was not significantly different from that of native (Fig. 2f, g). This observation may be consistent with the presence of functional secreted surfactant in the engineered lungs, with a concomitant reduction in alveolar surface tension^48^. Together, these data suggest that AEC2/FB/EC tri-culture supports lung scaffold repopulation with abundant AECs within organized alveolar-like units. These AEC2s exhibit characteristic molecular and morphometric features of native AEC2s^49^, and show evidence of mechanically relevant surfactant secretion. However, at this stage of culture, these AEC2s demonstrate minimal evidence of differentiation to the mature, squamous AEC1s required for gas exchange.

### Single-cell RNAseq demonstrates a pro-fibrotic signature in AEC2/FB co-cultured AEC2s

**Fig. 3:**
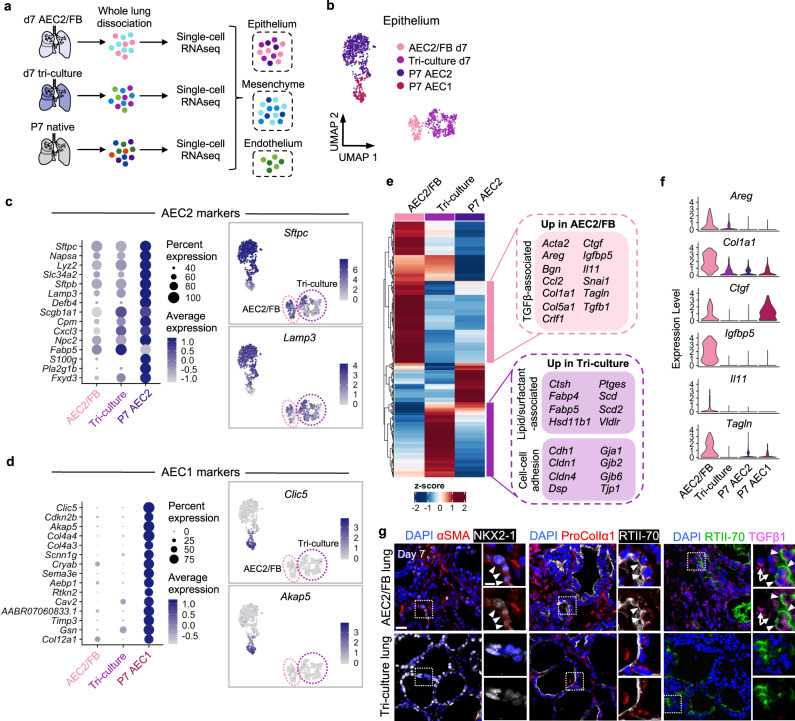
scRNAseq demonstrates a pro-fibrotic signature in AEC2/FB co-culture lung AEC2s. **a** Schematic of scRNAseq analysis of engineered and P7 native rat lungs. **b** Merged UMAP embedding of scRNAseq data for engineered epithelia and native P7 AECs. Populations have been merged into a single UMAP to facilitate phenotypic comparisons. **c**, **d** Dot plots (left) showing scaled expression and feature plots (right) showing normalized expression data projected onto the scRNAseq data from **b** for top AEC2 markers (**c**) and top AEC1 markers (**d**) in engineered lung and P7 native epithelial populations. **e** Heatmap of consensus DEGs across engineered epithelia and P7 native AEC2s, with select genes of interest indicated. **f** Violin plots of select TGFβ-associated genes identified from **e** to be enriched in AEC2/FB epithelium. **g** Immunostaining for protein expression of αSMA, ProColIα1, and TGFβ1 in d7 engineered lungs. Arrowheads, epithelial cells positive for these markers in AEC2/FB lung. Arrows, FBs positive for TGFβ1 in AEC2/FB lung. Scale bars, 25 μm; magnified region, 10 μm. Adjustments to contrast of individual color channels were performed identically across all samples.

We used scRNAseq of AEC2/FB and tri-culture engineered lungs at day 7 to further characterize epithelial cell phenotypes in each co-culture setting (Fig. 3a and Supplementary Fig. 7a, b). Native P7 rat lung populations served as comparison (Fig. 3b, Supplementary Fig. 1e–g, and Supplementary Data 1). The epithelium in both types of engineered lungs expressed canonical AEC2 genes, including *Sftpc*, *Slc34a2*, and *Lamp3*, among their top markers (Fig. 3c and Supplementary Data 1). Conversely, very little AEC1 marker expression was observed in either AEC2/FB or tri-culture epithelium at this stage of culture, consistent with the immunostaining and qRT-PCR analysis (Figs. 2a, b and 3d). (Interestingly, although the endothelium in tri-culture lungs demonstrated a shift toward native-like phenotypes following culture in lung scaffolds—with general [gCap] and shared capillary gene expression signatures being most prominent – ECs did not gain any additional aerocyte-like character by day 7 of culture in tri-culture lungs [Supplementary Fig. 7c]. Given the absence of AEC1s in these lungs, this observation may reflect the finding that AEC1s and aerocytes emerge synchronously in the developing lung^40,50^). Thus, these scRNAseq data confirm the AEC2-like phenotype of AEC2/FB and tri-culture engineered epithelia after 7 days of culture with CK + DCIR, albeit with somewhat decreased expression levels compared to P7 native.

However, closer examination of differentially expressed genes (DEGs) among AEC2/FB, tri-culture, and P7 AEC2s revealed key differences that distinguished co-culture AEC2s from their native counterparts (Fig. 3e and Supplementary Data 2). In particular, the AEC2/FB epithelium was uniquely enriched for several targets of transforming growth factor beta (TGFβ) signaling (including *Ctgf* and *Tagln*)^51^ and additional pro-fibrotic mediators (*Areg, Igfbp5, Il11*) that were negligibly expressed in native lung or tri-culture AEC2s (Fig. 3e, f)^52–54^. Immunostaining demonstrated protein expression of αSMA and procollagen Iα1 in a subset of co-cultured epithelium, a finding only rarely observed in tri-culture lungs (Fig. 3g). In addition, co-culture epithelium exhibited reduced expression of key epithelial transcription factor NKX2.1, as compared to tri-culture epithelium (Figs. 1d and 3g). TGFβ1 protein expression was widespread in both the epithelium and the FBs in co-culture lungs, but was nearly absent in tri-culture tissues (Fig. 3g).

In contrast, tri-culture AEC2s were enriched, as compared to native and co-culture AEC2s, for genes characteristic of lipid or surfactant synthesis (*Ctsh*, *Hsd11b1*, *Scd*)^13,55,56^ as well as for components of cell–cell junctions (*Cdh1*, *Cldn4*, *Tjp1*) (Fig. 3e). Collectively, these data demonstrate that while both co- and tri-culture epithelial populations retain some canonical AEC2 marker expression at the transcriptomic level, AEC2/FB co-culture in lung scaffolds—in the absence of endothelium—is also associated with abnormal AEC2 pro-fibrotic gene and protein expression. In contrast, tri-culture in the presence of ECs is associated with levels of fibrotic markers similar to native lung, combined with increased expression of markers associated with lipid metabolism in AEC2s. Such differences may contribute to the discrepancy in molecular and ultrastructural features previously noted in these AEC2 populations (Fig. 2c, d).

### Fibroblasts demonstrate reduced contractility and adopt additional niche-like features in the presence of ECs

**Fig. 4:**
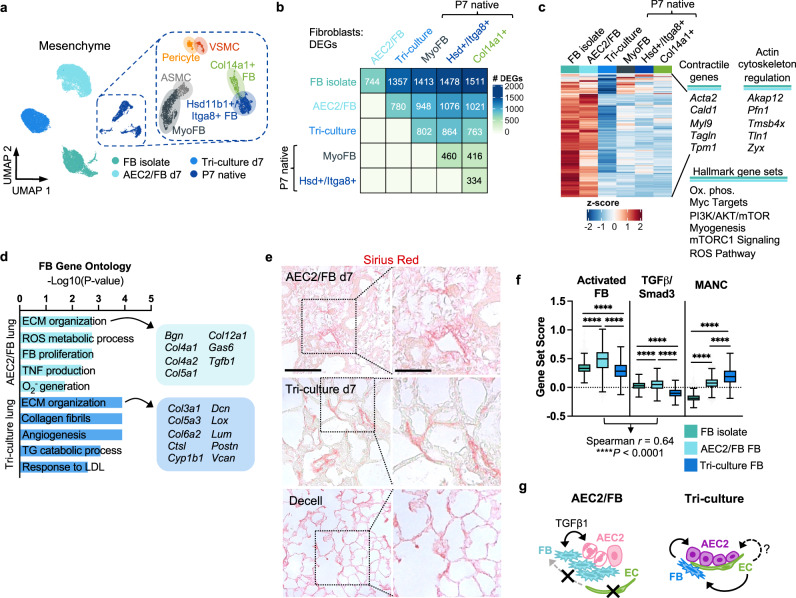
Endothelial co-culture is associated with mitigation of fibroblast activation and induction of niche-supportive features in neighboring mesenchyme. **a** Merged UMAP embedding of scRNAseq data for the FB isolate and engineered and native P7 mesenchyme. ASMC, airway smooth muscle cell. MyoFB, myofibroblast. VSMC, vascular smooth muscle cell. **b** Heatmap showing total numbers of differentially expressed genes between FB subsets. **c** Heatmap of genes enriched in both the FB isolate and in AEC2/FB FBs, as compared to tri-culture FBs, with associated pathway enrichment. P7 native FB populations are included for comparison. **d** Curated biological processes enriched in AEC2/FB and tri-culture FBs, respectively. DEGs were defined as those expressed in a minimum of 25% of cells with fold change ≥2 and adjusted *P* < 0.05. DEGs associated with “ECM organization” are indicated at right. **e** Representative images of Sirius Red staining for collagen in engineered and decellularized lungs. Scale bar, 100 μm. Magnified region, 50 μm. **f** Scoring of individual FBs for the indicated signatures. Scores > 0 indicate enriched expression compared to random gene sets. Correlation *r* between activated FB and TGFβ/Smad3 scores in co-culture FBs is indicated. MANC, mesenchymal alveolar niche cell. Boxplots: center indicates median, box limits indicate 1st and 3rd quartiles, whiskers indicate min and max (outliers not shown). Kruskal–Wallis test with Dunn’s post-test. ****P < 0.0001. **g** Proposed model of endothelial indirect support of AEC2s, whereby endothelial cells mitigate the contractile and activated features of neighboring FBs while inducing niche-supportive features in the mesenchyme.

Given the impact of tri-culture and the endothelium on AEC2 phenotype, we next asked whether FB phenotype was altered by the presence of endothelium within the engineered alveolar niche. P7 native rat lung mesenchymal populations served as benchmarks for single-cell analysis of engineered mesenchyme (Fig. 4a, Supplementary Fig. 2f, g, and Supplementary Data 3).

Comparison of DEGs among the cultured FB isolate, engineered lung FBs, and native lung FBs revealed that the polystyrene-expanded FBs were most different from native, and that the tri-culture FBs were most similar (Fig. 4b). For example, DEGs as compared to native Hsd11b1^+^/Itga8^+^ FBs numbered 1478 for the FB isolate, 1076 for AEC2/FB FBs, and only 864 for tri-culture FBs (Fig. 4b). Furthermore, whereas the polystyrene-expanded FBs and co-culture FBs were most similar to each other among the populations examined, tri-culture FBs were most similar to native Col14a1^+^ FBs, on the basis of overall transcriptomic signature, as well as by a specific Col14a1^+^ gene set score (Fig. 4b and Supplementary Fig. 7d). Thus, epithelial co-culture in lung scaffolds, and more so epithelial-endothelial tri-culture, shifted the cultured FB isolate back toward native FB phenotypes.

Under conditions of AEC2/FB co-culture, but not tri-culture, we found that FBs retained elevated expression of contractile markers (*Acta2*, *Myl9*), as seen in the cultured FB isolate; and were enriched for pathways (mTORC1 signaling, reactive oxygen species [ROS] metabolism) and matrix-related molecules (*Tgfb1, Col5a1, Col12a1*) that are characteristic of FBs in pulmonary fibrosis, but that are minimally expressed in native lung FBs (Fig. 4c, d, Supplementary Fig. 7e, f, and Supplementary Data 3)^57,58^. Indeed, by Sirius red staining, AEC2/FB lungs exhibited what appeared to be widespread, disorganized collagen deposition and/or remodeling, with accompanying disruption of the alveolar meshwork (Fig. 4e). As indicated by scoring for an unbiased activation signature^58^, co-culture FBs were significantly more activated than both the starting FB isolate and tri-culture FBs, in a manner moderately correlated with TGFβ responsiveness (Fig. 4f and Supplementary Fig. 7g). Thus, in the absence of endothelium, co-cultured FBs demonstrate evidence of phenotypic alterations related to TGFβ signaling, including increased contractility and matrix remodeling.

The Col14a1^+^ FB-like phenotype of tri-culture FBs (Fig. 4b and Supplementary Fig. 7d) is notable given the similarity of Col14a1^+^ FBs in our native dataset to the matrix-producing, AEC2-supportive mesenchymal alveolar niche cells (MANCs) proposed by Zepp and colleagues (Supplementary Fig. 7h)^27^. In addition to the *Axin2*^+^/*Pdgfra*^+^ MANC signature (Fig. 4f and Supplementary Fig. 7i), tri-culture FBs were enriched for genes associated with angiogenesis and lipid handling (Fig. 4d and Supplementary Fig. 7j). The latter suggests a possible role for tri-culture FBs in supporting AEC2 surfactant synthesis in bioengineered lungs^59^. ECM genes that were upregulated in tri-culture FBs, relative to AEC2/FB co-cultured mesenchyme, included proteoglycans that are critical for proper collagen fibril assembly (*Dcn*, *Lum*), as well as a basement membrane collagen (*Col6a2*) that is necessary for normal alveolar formation (Fig. 4d)^60^. Sirius red staining of tri-culture lungs revealed well-defined collagen within the alveolar septa with airspace preservation – albeit with some alteration of the original alveolar network (Fig. 4e). Together, these data suggest that ECs may modulate FB phenotype to maintain FB quiescence and to promote alveolar mesenchymal phenotypes (Fig. 4g).

### Endothelial cells shift fibroblast-epithelial communication patterns

**Fig. 5:**
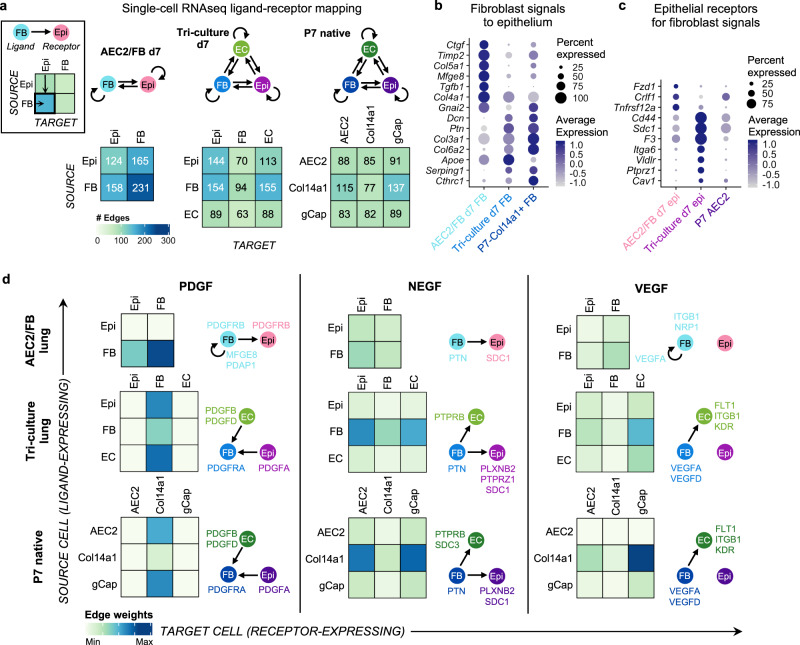
Endothelial co-culture shifts fibroblast-AEC2 communication. **a** Schematic of signaling networks in d7 engineered lungs and amongst AEC2s, Col14a1^+^ FBs (Col14a1) and general capillary endothelial cells (gCap) in P7 native lung. Numbers indicate the total number of putative ligand-receptor (LR) pairs expressed along each cell–cell vector, as indicated in the heatmap key at left. **b** Dot plot of top differentially expressed FB ligands with epithelial cognate receptors in d7 engineered lungs. **c** Dot plot of top differentially expressed epithelial receptors with FB cognate ligands in d7 engineered lungs. **d** Heatmaps of summed edge weights for all LR pairs along the given cell–cell vectors within each of the indicated signaling pathways, in d7 engineered lungs and amongst native cell populations. Color scale for edge weights applies to each pathway individually. Schematics to the right of each panel indicate select top LR pairs contributing to the predominant signaling vectors.

To further investigate the changes introduced to the engineered alveolar niche by the inclusion of ECs, we mapped a known database of ligand-receptor (LR) pairs to the scRNAseq data to generate putative signaling networks within each engineered lung (Fig. 5a and Supplementary Data 4)^61^. As a native comparator, we focused on the LR pairs among P7 native AEC2s and select other cell types in the native alveolar niche. For the FB population, we focused on native Col14a1^+^ FBs, which are the population most similar to MANCs by scRNAseq (Supplementary Fig. 7h). For the EC population, we focused on native general capillary (gCap) ECs, which are the capillary EC type likely to be in closest proximity to AEC2s in the native alveolus (Fig. 5a and Supplementary Data 4)^40^.

LR pairs in the AEC2/FB lung, which lacked ECs, demonstrated a preponderance of autocrine FB signaling (231 edges) (Fig. 5a). In contrast, FB autocrine signaling was dramatically reduced in tri-culture lung (94 edges) to a level more comparable to that observed between Col14a1^+^ FBs in the native lung (77 edges; Fig. 5a). Notably, the ECs in tri-culture lung were involved in numerous paracrine signaling exchanges (at least 63 LR pairs per cell–cell vector) with both the epithelium and the mesenchyme, a pattern that mirrors the numerous signaling exchanges between gCap cells and both AEC2s and Col14a1^+^ FBs, respectively, in native lung (Fig. 5a). In fact, the overall distribution of signaling edges in tri-culture lung was much more similar in pattern and magnitude to that observed amongst the examined native lung populations (Fig. 5a). This analysis suggests that the endothelium has integral signaling roles within the native alveolus, and that incorporating ECs into the engineered system introduces global changes to the signaling landscape.

In AEC2/FB co-cultured lung, top differentially expressed FB ligands with capacity to affect both AEC2s and FBs included pro-fibrotic cytokines *Ctgf* and *Tgfb1* (Fig. 5b and Supplementary Data 4). In contrast, within tri-culture lung containing ECs, FBs were enriched for ligands that were notably shared by the native Col14a1^+^ FBs, including antifibrotic modulators *Dcn*^62^ and *Cthrc1*^63^, as well as *Ptn* (pleiotrophin), a PDGF-induced growth factor associated with stem cell expansion and tissue regeneration across several organ systems (Fig. 5b)^64–67^. In tri-culture lung, as compared to AEC2/FB lung, enriched epithelial receptors that had cognate FB ligands included the pleiotrophin receptor *Ptprz1*, as well as *Cd44*, which has been proposed to label a subpopulation of particularly regenerative AEC2s lying in close proximity to capillaries (Fig. 5c and Supplementary Data 4)^68^. Interestingly, many of these upregulated epithelial receptors within the tri-culture system also had endothelial cognate ligands (Supplementary Data 4). These data show that AEC2/FB co-culture lungs harbor FBs that send pro-fibrotic signals to both FBs and AEC2s, and that this pro-fibrotic signaling is attenuated when ECs are added to the system. Furthermore, in the tri-culture setting, AEC2s express markers that are associated with epithelial renewal, which are minimally expressed in the simpler co-culture model lacking ECs.

To understand broader signaling milieux within the engineered lungs, we examined several key signaling pathways, and compared their expression patterns to those in native P7 lung (Fig. 5d). We observed that platelet-derived growth factor (PDGF) signaling demonstrated a directional shift in the predominant signaling vector with the addition of ECs to the engineered lung. Specifically, in AEC2/FB lung, FBs were the dominant source of PDGF ligands, signaling to both the epithelium as well as to FBs. In contrast, when ECs were added to the tri-culture system, then the predominant PDGF signaling vectors were directed to FBs, in the form of *Pdgfa* ligand from AEC2s, and *Pdgfb* and *Pdgfd* ligands from ECs (Fig. 5d). This shift was coincident with the upregulation of *Pdgfra* expression in tri-culture FBs (Supplementary Fig. 7i), and is an overall signaling pattern more closely matching that of native P7 lung tissue (Fig. 5d).

Other pathways, including the neurite growth-promoting factors (NEGF) family cytokines *Ptn* and *Mdk* (midkine), were enriched in the presence of ECs in tri-culture lung, similar to expression patterns in native lung (Fig. 5d). The vascular endothelial growth factor (VEGF) pathway, which was minimally expressed in AEC2/FB lung, was reintroduced in tri-culture lung with the addition of ECs. Dominant receptors on ECs in the tri-culture lung were similar to those of native lung (Fig. 5d). Taken together, these data show how the presence of ECs impacts gene expression by both the epithelium and the mesenchyme, and how the inclusion of vascular endothelium within the tri-culture system produces a higher fidelity signaling milieu.

### Wnt and FGF withdrawal is a modest cue for AEC2 differentiation to AEC1s

We next turned our attention toward the goal of deriving squamous AEC1s within the tri-culture engineered alveoli. While tri-culture lungs exhibited large numbers of AEC2s, few to no AEC1s were appreciated in the tri-culture system at day 7 (Figs. 2a, b and 3d). Prior evidence suggests that CK + DCI supplementation supports long-term maintenance of hPSC-derived AEC2s, but does not support their further differentiation into AEC1s^20^. We hypothesized that removal of pro-proliferative factors, following epithelial expansion, may permit further differentiation of the engrafted AEC2s. In particular, withdrawing the Wnt agonist CHIR and the FGFR2 agonist KGF (together, “CK”) should remove key signals favoring AEC2 proliferation and fate maintenance in the tri-culture system, and might permit AEC2 differentiation to AEC1s^20,25,69–72^. We confirmed key roles for CHIR and KGF in supporting AEC2 growth within our tri-culture system using an alveolosphere assay, in which we evaluated the effect of excluding each CK + DCIR medium component on AEC2/FB/EC alveolosphere formation (Supplementary Fig. 8a). Excluding either CHIR or KGF resulted in a significant reduction in both the number and size of alveolospheres that formed over 7 days (Supplementary Fig. 8b, c).

**Fig. 6:**
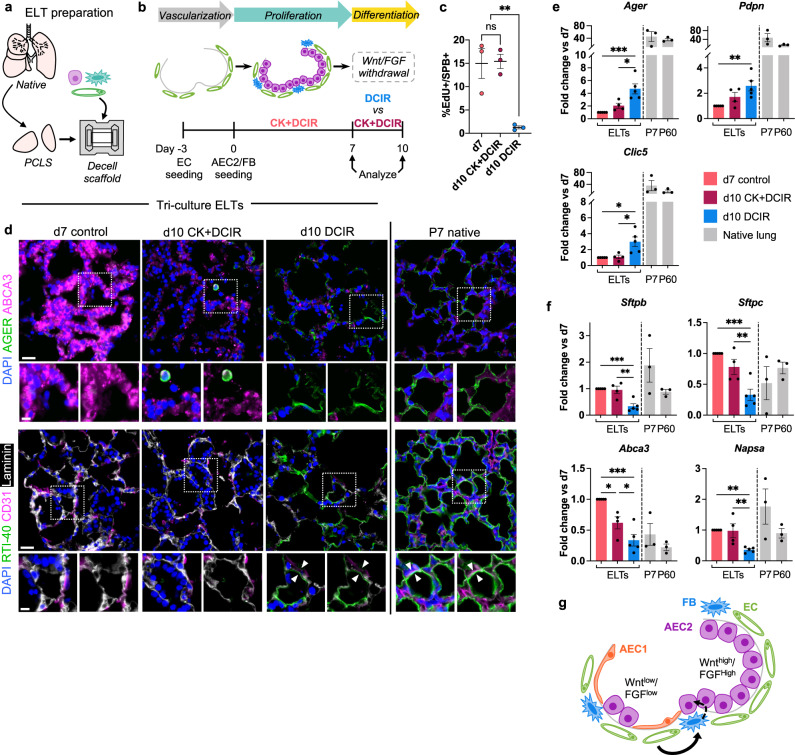
Wnt/FGF withdrawal is a modest cue for AEC2 differentiation. **a** Schematic of tri-culture engineered lung tissue (ELT) preparation. PCLS, precision-cut lung slice. **b** Timeline of experiment examining effects of CHIR and KGF removal on AEC2 differentiation to AEC1s in tri-culture ELTs. **c** Quantification of EdU^+^ AEC2s in ELTs via immunostaining; *n* = 3. **d** Immunostaining for AEC1 proteins AGER and RTI-40 in ELTs, with P7 native rat lung shown for comparison. Arrowheads indicate close apposition of an RTI-40^+^ AEC1 with a CD31^+^ EC. Scale bars: main image, 25 μm; magnified region, 10 μm. qRT-PCR of AEC1 (**e**) and AEC2 (**f**) gene expression in ELTs. Native gene expression is normalized to the average expression in d7 tissues and shown for approximate comparison only. d10 CK + DCIR: *n* = 4; d7, d10 DCIR: *n* = 5; native: *n* = 3. **g** Proposed role for Wnt and FGF in regulating epithelial fate within the alveolus. **c**, **e**, **f** Error bars: mean ± SEM. One-way ANOVA with Holm–Sidak’s multiple comparisons tests. ns not significant, *P < 0.05, **P < 0.01, ***P < 0.001.

To test both biochemical and mechanical cues for AEC1 differentiation within cultured lung scaffolds, we developed a platform to allow precise application of mechanical strain to 3D lung intact extracellular matrices, by adapting our prior work with engineered heart tissues^73,74^. We generated small-scale engineered lungs tissues (ELTs) by reseeding acellular precision-cut lung slice matrices (Fig. 6a)^75^. ELTs were seeded with ECs followed by AEC2s and FBs, and cultured for an initial 7-day proliferation phase, analogous to the tri-culture lungs described above. Thereafter, we removed CK (leaving behind the “DCIR” medium) for an ensuing 3 days (“d10 DCIR”) that comprised the differentiation phase of culture (Fig. 6b). After 3 days of CK withdrawal, quantification of 5-ethynyl-2´-deoxyuridine (EdU) incorporation confirmed a significant decrease in AEC2 proliferation (Fig. 6c). This was accompanied by the striking appearance of many engineered alveoli that were lined with squamous-appearing, AGER^+^ or RTI-40 (podoplanin)^+^ AEC1-like cells at day 10, with a microscopic tissue appearance somewhat approaching that of native lung (Fig. 6d). We also observed closely apposed RTI-40^+^ AEC1 and CD31^+^ EC cellular processes at day 10, mimicking the apposition that is characteristic of blood-gas barrier formation (Fig. 6d)^44^. qRT-PCR revealed a modest but significant increase in AEC1 gene expression at day 10 after 3 days of CK withdrawal, with a concomitant decrease in AEC2 gene expression, as compared to day 7 control ELTs (Fig. 6e, f).

In contrast, ELTs that were cultured from days 7 to 10 with CK + DCIR (“d10 CK + DCIR”) exhibited no change in AEC2 proliferation, and no significant evidence of differentiation to AEC1s as compared to day 7 ELTs (Fig. 6c–f). This implies that the partial AEC1 differentiation that was observed with CK withdrawal was not simply a consequence of increased time in culture, but rather a consequence of the removal of agonism for the Wnt and FGF signaling pathways. Together, these data suggest that Wnt and FGF agonism, via the CK factors, promotes the maintenance of AEC2 fate. However, as suggested by the persistence of clusters of AEC2s (Fig. 6d) and the only modest increase in AEC1 gene expression (Fig. 6e) following CK withdrawal, a low-Wnt and -FGF state is an insufficient stimulus for robust and widespread AEC1 differentiation (Fig. 6g).

### Tidal strain synergizes with Wnt/FGF withdrawal to promote the AEC1 fate

**Fig. 7:**
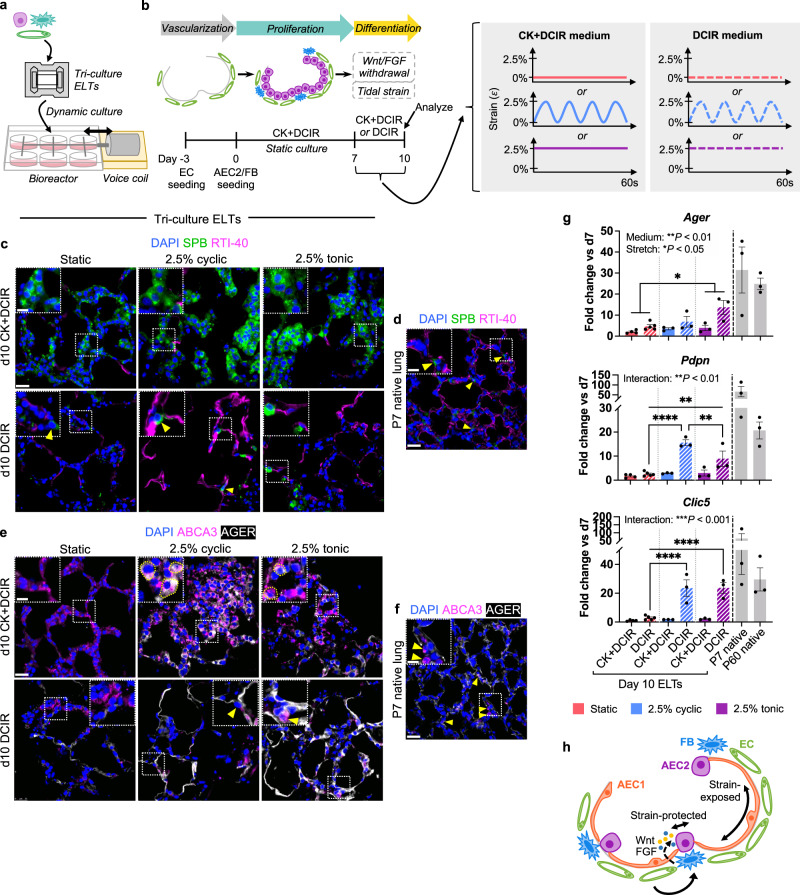
Tidal strain synergizes with biochemical cues to promote the AEC1 fate in engineered lung. **a** Schematic of bioreactor for engineered lung tissue (ELT) dynamic culture. **b** Timeline of experiment examining effects of CHIR and KGF withdrawal, with or without applied tidal strain, on AEC2 differentiation in tri-culture ELTs. **c**–**f** Immunostaining for AEC2 (SPB, ABCA3) and AEC1 (RTI-40, AGER) markers in tri-culture ELTs at d10 (**c**, **e**) or in P7 native lung (**d**, **f**). Yellow arrowheads, AEC2s localized to alveolar corners. Yellow dotted outlines, AGER^+^/ABCA3^+^ cells. Scale bars: main image, 25 μm; magnified region, 10 μm. Note that imaging parameters used for native tissues in **d**, **f** differ from those used in the corresponding ELTs, as higher exposures were generally required for native lung samples to visualize the proteins. **g** qRT-PCR of AEC1 gene expression in d10 ELTs, normalized to their respective d7 control tissues. Data for static controls are repeated from Fig. 6e. Native gene expression is normalized to the average expression in d7 tissues and shown for approximate comparison only. Static/CK + DCIR: *n* = 4; static/DCIR: *n* = 5; all other conditions: *n* = 3. Error bars: mean ± SEM. Text indicates significance by two-way ANOVA for medium main effect (“medium”), stretch main effect (“stretch”), and interaction between medium/stretch (“interaction”). *P* values for annotated pairwise comparisons were calculated via Holm-Sidak’s multiple comparisons tests. *P < 0.05, **P < 0.01, ****P < 0.0001. **h** Proposed role for Wnt/FGF and tidal strain in regulating epithelial fate within the alveolus.

Mechanical forces exerted on cells as a consequence of breathing are important regulators of lung epithelial phenotype. In particular, accumulating evidence points to activation of the mechanosensitive YAP/TAZ pathway as requisite for the AEC1 fate^76,77^. The observation that AEC1s are distended more by lung inflation than are their AEC2 neighbors^78^ similarly suggests that AEC1s experience a unique mechanical environment within the alveolus. Hence, we tested the application of native-like mechanical forces—specifically, the mechanical strain associated with tidal ventilation—as an alternative cue for AEC2 differentiation to AEC1s in engineered alveoli. ELTs were repopulated with AEC2s, FBs, and ECs via static culture through day 7 in the presence of CK + DCIR medium. Tri-culture ELTs were then transferred to a cyclic strain bioreactor^74^ for 3 additional days of culture, under either 2.5% cyclic (4 cycles/min) or 2.5% tonic uniaxial strain (to mimic the strain associated with tidal breathing^79^), in the presence of either CK + DCIR or DCIR medium (Fig. 7a, b). ELTs that were previously cultured through day 10 under static conditions served as no-strain controls.

After 3 days of applied cyclic or tonic strain in the bioreactor, ELTs cultured with CK + DCIR medium through day 10 contained epithelial clusters and alveolar-like structures that were lined with cuboidal AEC2 monolayers (Fig. 7c–f and Supplementary Fig. 8d–g). Only a few flattened cells were observed that expressed the AEC1 markers RTI-40 or AGER (Fig. 7c–f), and AEC1 gene expression by whole-tissue qRT-PCR was very low (Fig. 7g). However, tissues cultured with CK + DCIR under strain contained multiple cuboidal AGER^+^/ABCA3^+^ cells (Fig. 7e), which may represent AEC2s in an intermediate state of differentiation toward an AEC1 phenotype.

In contrast, ELTs cultured with DCIR (i.e. lacking CK) under cyclic or tonic strain for 3 days comprised alveolar-like structures that were generally lined by squamous AEC1-like cells (Fig. 7c–f). RTI-40 and AGER protein expression was noticeably brighter and more widespread in tissues exposed to tidal-magnitude strain in DCIR medium, as compared to tissues that were cultured statically in DCIR medium (Fig. 7c, e). Although AEC2 gene expression was significantly reduced following the removal of CHIR and KGF for the final 3 days of culture (Supplementary Fig. 8h), scattered AEC2s remained that were often localized to the alveolar corners, which is their typical location in the native lung (Fig. 7c–f and Supplementary Fig. 8d–g)^80^. qRT-PCR confirmed the immunofluorescent staining, and demonstrated a significant synergy between stretch and CK withdrawal in promoting AEC1 differentiation (Fig. 7g). Indeed, AEC1 gene expression approached native tissue levels in DCIR-treated, stretched ELTs after 3 days of tidal-level strain (Fig. 7g). On a more subtle level, the effect of cyclic stretch versus tonic stretch appeared to vary by marker, which suggests that the stretch pattern may confer differences in cell function or molecular state that are not captured by the analysis performed here (Fig. 7c, e, g). Taken together, these findings suggest that mechanical stretch alone is unable to effect mature, squamous AEC1 differentiation when high levels of Wnt and FGF agonism are present. However, a Wnt- and FGF-low environment synergizes with tidal-level strain to promote extensive AEC2 differentiation to AEC1s within the engineered lung tissues (Fig. 7h). The engineered lung system, containing discrete and controllable cues and cell populations, allows the dissection of these pivotal differentiation cues.

## Discussion

Our results demonstrate the de novo formation of alveolar epithelium comprising AEC2s and squamous AEC1s within networks of epithelial-mesenchymal-endothelial alveolar-like units in engineered lung. This epithelial organ population has not been observed during the previous decade of whole lung engineering efforts reported in the literature—neither in the fidelity of epithelial phenotype, nor in the degree of recapitulation of the surrounding alveolus^3,4,7–11^. We developed a rational, two-phase culture strategy combining multi-lineage co-culture, soluble growth and maturation factors, and mechanical strain to first promote AEC2 expansion, and then to drive the differentiation of AEC1s, in situ in decellularized lung scaffolds. Through investigations with this engineered co-culture system—which uniquely uses defined culture conditions and a physiologically-relevant 3D culture substrate—we also highlighted previously underappreciated roles for vascular endothelium and physiological strain within the alveolar niche.

A significant hurdle for lung alveolar engineering—and for lung cellular therapies—has been maintaining alveolar epithelial phenotypes among cells that engraft in injured lungs (or in denuded scaffolds)^8,81,82^. One study found a portion of cultured AEC2s, but not freshly isolated AEC2s, to adopt a dysplastic keratin 5^+^ fate following transplantation into injured lungs^82^. Another found that AEC2s that could differentiate in organoid culture failed to differentiate into AEC1s in vivo, even weeks following AEC2 transplantation^81^. Together, these observations suggest that the regenerative potential of AEC2s depends on the conditions of their microenvironment—and conversely, that an altered niche, including the conditions of in vitro culture, may interfere with AEC2 behavior. Indeed, it is known that activation of pro-fibrotic signaling^83^ or ectopic expression of proximal airway pathways^26^ in distal lung FBs disrupts AEC2 progenitor function. However, bolstering the niche at the time of epithelial cell delivery—such as via simultaneous inhibition of pro-fibrotic FB signaling^28^ or concomitant delivery of FBs and ECs^84^—has been shown to enhance alveolar epithelial regeneration in injured lungs.

In this work, we found that while neighboring stromal cells did not impact initial epithelial engraftment, they significantly altered subsequent patterns of tissue repopulation. Specifically, native-like AEC2 phenotype, alveolar structure, and cellular signaling patterns in the first phase of engineered lung culture were better recapitulated via epithelial-mesenchymal-endothelial tri-culture rather than by a more simplified system. This is a notable finding given the recent pursuit of AEC2 monocultures for cell expansion and therapy^17–20,82,85^. Our data suggest that FBs are essential for AEC2 growth in our system, but that ECs, in concert with FBs, improve tissue organization and mitigate FB pro-fibrotic signaling. Indeed, disrupting capillary development and normal epithelial-endothelial signaling in early lung development has been shown to impair alveolar morphogenesis^50,86–89^. Healthy ECs are also known to help protect the epithelium while exerting antifibrotic effects on neighboring FBs to enhance lung injury resolution in settings of pulmonary fibrosis^28,83,90,91^.

We also noted an interesting shift in FB phenotype in the tri-culture system away from an activated and pro-fibrotic state, toward that of the *Axin2*^+^/*Pdgfra*^*+*^ MANCs that were recently identified as a critical component of the AEC2 niche^27^. Additionally, FBs in the tri-culture system showed upregulation of lipid-related genes, which suggests a possible role in supporting surfactant secretion in the engineered system. Given that the only difference between tri-culture and AEC2/FB co-culture lungs is the presence or absence of endothelium, these observations suggest that FBs may rely on signals from neighboring ECs to maintain a niche-supportive state. In a notable similarity, a recent report found that EC co-culture was sufficient to induce Axin2 expression in sorted Axin2^−^ FBs, in turn enhancing FB niche support of epithelial branching in the mammary gland^92^. While the specific mechanism(s) of endothelial-mesenchymal crosstalk in our system remain to be elucidated, these data together emphasize that microenvironmental cues—not just inherent cellular identity—contribute to the capacity of FBs to fulfill a niche role.

We also found that tidal-level strain synergized with Wnt/FGF withdrawal to promote AEC2 differentiation to AEC1s within cultured lung tissue in just three days. Our finding that stretch is only an effective stimulus for AEC1 differentiation in the absence of CHIR and KGF may seem contradictory to prior reports, which have consistently demonstrated AEC1-like differentiation in the setting of stiff substrates or tonic strain^15,18,34,69,93,94^. However, it is noteworthy that such studies have typically been conducted under conditions that a priori would have promoted the loss of a differentiated AEC2 phenotype: i.e. in 2D culture^95^, on a fibronectin-coated surface^96^, or in serum-containing medium^97^. Rather, when cultured in the absence of serum and on a physiological lung matrix substrate, AEC2s show a dominant role for Wnt and/or FGF signaling in maintaining their AEC2-like fate. Even in the presence of cyclic strain resembling breathing, the presence of Wnt and FGF agonism largely prevented the differentiation to an AEC1-like state in the engineered lung system. This observation is consistent with recent reports that FGFR2 expression restricts alveolar epithelium to the AEC2 fate in neonatal lung^71,98^. This apparent relationship of soluble to mechanical signals could also shed some light on previous observations that genetic YAP activation in AEC2s or in alveolar progenitors (in effect, activation of some of the downstream signaling induced by mechanical stretch, via a molecular approach) is ineffective at inducing full differentiation of squamous AEC1s^76,77,99,100^. It may be that local variation in strain levels within the alveolus^78^ acts in tandem with short-range FB-derived Wnts^25^ and AEC2-specific FGFR2 expression^34,71,98^ to determine epithelial differentiation into AEC2 versus AEC1 types.

The current study, by focusing on recapitulating cellular-level epithelial phenotypes in vitro in engineered lung, in particular squamous AEC1s, addresses a significant stumbling block in the path toward regenerating functional lung tissue. Our choice of cell populations was necessarily constrained by the large cell numbers required for whole organ cultures—which led us to use easily-isolated neonatal AEC2s and low-passage FBs and ECs that were exposed to hard plastic for expansion prior to seeding into the lung. The use of non-age-matched ECs might also have influenced the results in this study, although the profound impact of endothelium in improving neighboring cellular phenotypes and nascent tissue organization speaks to a critical role for endothelium within the alveolar niche, notwithstanding the developmentally older age of the cells used here. The impact of alveologenic, neonatal ECs on tissue repopulation would be an interesting area for future investigation. Because recent observations suggest that neonatal AEC2s exhibit differences in fate regulation compared to adult AEC2s^71,77^, additional optimization may be needed to adopt the strategies for directed expansion and differentiation used in this work, for organ repopulation with adult or hPSC-derived AEC2s. Nevertheless, our data emphasize the remarkable plasticity of cells in in vitro culture, as well as the potential for variables within the engineered microenvironment to be tools for guiding cell fate and behavior toward the reconstitution of a complex tissue. The rigor of phenotypic interrogation in this study – with multiple modalities of comparison to native lung tissue – is important for the lung engineering field, while simultaneously underscoring the high bar for success that native verisimilitude presents. Further functional assessments and evaluation of the long-term durability of the engineered tissue will be essential future steps to evaluate appropriateness for transplantation. Integrating the strategies presented here with previous advances in bioengineered lung microvascular regeneration^5,6^, and incorporating yet additional niche complexity, including alveolar macrophages and an air-liquid interface, will likely be additional important steps toward improving engineered lung phenotype.

## Methods

### Animals

All animal work was performed in accordance with AAALAC guidelines and was approved by the Yale Institutional Animal Care and Use Committee. Animal husbandry and veterinary care were provided by the Yale Animal Resources Center. Animals were group housed, provided clean bedding and *ad libitum* water and food, and housed under controlled temperature and humidity with a 12 h light/dark cycle. Wild-type Sprague-Dawley rats were purchased from Charles River Laboratories and used for decellularized lung scaffold preparation and primary AEC2 and fibroblast isolations. GFP^+^ AEC2s were isolated from the lungs of SD-Tg(CAG-*EGFP*)4Osb (*EGFP*^+^) rats, in which *EGFP* is driven by the ubiquitous CAG promoter (originally from Dr. Masaru Okabe, Osaka University, Japan)^101^.

### Isolation of rat AEC2s

Rat alveolar epithelial type 2 cells (AEC2s) were isolated from 7–9-day-old wild-type or *EGFP*^+^ Sprague-Dawley rat pups as previously described^8^, with slight modifications. In most cases, 5–10 pups were randomly selected from a single litter without bias for males or females. For a given experiment, each biological replicate used an independent AEC2 isolate. Briefly, pups were euthanized via intraperitoneal (IP) injection of sodium pentobarbital (150 mg/kg). The thorax was entered and the lungs were flushed of blood by intracardiac injection of 100 U/mL heparin and 0.01 mg/mL sodium nitroprusside in PBS. The trachea was cannulated with a 24 G, 0.75-inch intravenous catheter (BD). The lungs were inflated with dispase (2 U/mL; Roche) in Dulbecco’s modified Eagle’s medium (DMEM) with 20% HEPES-buffered saline (50 mM HEPES and 150 mM NaCl, pH 7.4 in dH_2_O), followed by 1% low-melting-point (LMP) agarose. The agarose was gelled under ice, and then the lungs were extracted and incubated in additional dispase solution for 45 min at room temperature. The distal lung tissue was separated from the mainstem bronchi and transferred to a Petri dish containing DMEM with 1% HEPES and 100 U/mL DNase I (Worthington). The tissue was then dissociated with a blunt bone marrow aspiration needle, 1 mM EDTA was added, and the cell suspension was sequentially filtered through 100- and 40-μm strainers, and 20-μm nylon mesh. The cells were labeled with RTII-70 antibody^102^ in buffer (Hank’s balanced salt solution [HBSS] with 2 mM EDTA and 0.5% BSA), tagged with anti-mouse IgG microbeads (Miltenyi), and magnetically sorted via MS columns (Miltenyi). Freshly isolated cells were used immediately in all experiments, without intervening culture.

### Isolation and culture of rat lung fibroblasts

Neonatal rat lung fibroblasts (FBs) were isolated from 7–9-day-old Sprague-Dawley rat pups using a method similar to that described by Bruce and Honaker^103^. For each isolation, 5–10 pups were randomly selected from a single litter without bias for males or females. For whole lung experiments, each replicate used cells from an independent FB isolation. Pups were euthanized, the chest was entered, and lungs were flushed of blood as described for the isolation of AEC2s and excised *en bloc*. Following removal of the heart and hilar structures, the lungs were minced and digested in 0.5 mg/mL trypsin (Gibco) with 0.3 mg/mL collagenase type I (Worthington) in HBSS for 2 h in a shaking water bath at 37 °C, with trituration every 15 min. One hour into the digestion, the cells in suspension were removed and mixed 1:1 with DMEM containing 10% FBS. Fresh enzyme solution with 100 U/mL DNase I was added to the remaining tissue pieces, and incubation continued for 1 h. At the end of the incubation, any remaining tissue pieces were dissociated with a blunt bone marrow aspiration needle and mixed 1:1 with DMEM with serum. The combined cell suspensions were filtered through 100- and 70-μm strainers, centrifuged at 400 × *g* for 10 min at 4 °C, and resuspended in HBSS. The cells were plated at 30–40 million cells per T75 cell culture flask with the balance in volume to 15 mL per flask made up by DMEM with 10% FBS and 1% penicillin/streptomycin (P/S, final serum concentration 8%). The flasks were incubated at 37 °C for 60 min, then aspirated of non-adherent cells, rinsed with PBS, and replenished with fresh medium (DMEM + 10% FBS + 1% P/S). On day 1 after plating, the flasks were rinsed once more with PBS and replenished with fresh culture medium. For whole lung cultures, freshly isolated FBs were cultured approximately 72 h in a humidified incubator at 37 °C and 5% CO_2_, with a medium change after 24 h, and used at passage 1. For alveolosphere and engineered lung tissue (ELT) cultures, FBs were used at passage 1–2.

### Endothelial cell culture

Primary rat lung microvascular endothelial cells (RLMVEC; isolated from 4–6 week-old male Sprague-Dawley rats, VEC Technologies) were cultured on fibronectin (1 μg/cm^2^)-coated flasks in MCDB-131 Complete medium with serum (VEC Technologies) and used at passage 5–6. Cells were cultured in a humidified incubator at 37 °C and 5% CO_2_, with medium changes every other day.

### Whole lung decellularization

Decellularized whole lung scaffolds were prepared from the lungs of 300–350 g male Sprague-Dawley rats (approximately 8–12 weeks of age). Animals were anesthetized via IP injection of ketamine (75 mg/kg) and xylazine (5 mg/kg), followed by IP injection of heparin (400 U/kg). The thorax was entered and lungs were cannulated in situ via the trachea and pulmonary artery (PA) with 1/16-inch barbed fittings (Cole Parmer), then cleared of blood via PA perfusion with 100 U/mL heparin and 0.01 mg/mL sodium nitroprusside in PBS with simultaneous manual ventilation of the airways via 10 mL syringe. Lungs and heart were extracted *en bloc* and decellularized with a Triton X-100/sodium deoxycholate (SDC)-based protocol as previously described^36^. Briefly, lungs were rinsed with antibiotics/antimycotics (10% P/S, 4% amphotericin-B, 0.4% gentamicin in PBS with Ca^2+^ and Mg^2+^) followed by perfusion with 0.0035% Triton X-100. Lungs were inflated via the trachea with benzonase endonuclease (20 U/mL) and incubated for 30 min, then rinsed via the PA with 1 M NaCl and a gradient of sodium deoxycholate solutions (0.01%, 0.05%, 0.1%). Next, lungs were perfused with 20 U/mL benzonase and incubated for 60 min. Finally, lungs were perfused with 0.5% Triton X-100 and rinsed extensively with PBS. Decellularized scaffolds were stored in antibiotics/antimycotics solution for up to 30 days at 4 °C before use.

### Engineered whole lung culture and harvest

For engineered whole lung cultures, lung scaffolds were mounted in our previously described bioreactor system with oxygenation via hollow-fiber cartridge^43^. For endothelial cell-seeded lungs, scaffolds were pre-seeded on day −3 with 38.0 ± 1.4 M RLMVEC via the pulmonary veins (PV) and 38.8 ± 1.4 M RLMVEC via the PA as previously described^41^, and cultured for 3 days in MCDB-131 complete medium with 2% FBS, 1% P/S, and 0.1% gentamicin with 20 mL/min perfusion via the PA, in an incubator at 37 °C with 5% CO_2_. On day 0 of culture, the bioreactor medium was replaced with CK + DCIR medium and scaffolds were seeded with 40 M AEC2s with or without 36.4 ± 1.8 M FB via injection into the trachea. Following both endothelial and epithelial cell seedings, the lung was allowed to sit statically for 90 min at 37 °C. PA perfusion was then resumed at 2 mL/min, and ramped up to 20 mL/min over the course of 3 h. CK + DCIR medium consists of epithelial base medium (50% DMEM (Gibco), 50% F-12 Nutrient Mixture (Gibco), 15 mM HEPES (Corning), 4 mM L-glutamine (Gibco), 1% P/S (Gibco), 0.1% gentamicin (Gemini Bio), 10 μg/mL insulin (Sigma), 5 μg/mL transferrin (Sigma), 0.1% BSA Fraction V (Gemini Bio)) supplemented with CK + DCIR additives (3 μM CHIR99021 [CHIR; PeproTech], 10 ng/mL keratinocyte growth factor [KGF; PeproTech], 50 nM dexamethasone [Sigma], 0.1 mM 8-Bromo cAMP [Sigma], 0.1 mM 3-Isobutyl-1-methylxanthine [IBMX; Sigma], 0.01 μM retinoic acid [Sigma]) (adapted from refs. ^20,104^). On day 3 of culture following epithelial seeding, the right lower lobe was tied off and removed. The lobe was divided for qRT-PCR analysis (snap frozen) and histological analysis (injected to inflate with 10% neutral-buffered formalin (NBF) and fixed for 4 h). Lung PA perfusion was decreased to 14 mL/min and culture continued for an additional 4 days. Throughout culture, partial media changes were performed daily, titrating to maintain medium glucose >70 mg/dL and lactate <10 mM (monitored using glucose test strips [GlucCell] and lactate test strips [Nova Biomedical]). The remaining engineered lung lobes were harvested on day 7 of culture following epithelial seeding. Lobes were either snap frozen; inflated with 10% NBF at 10–15 cm cmH_2_O via the trachea, then allowed to fix for 4 h prior to histological processing; or dissociated for scRNAseq (see details below).

### Pressure-volume curves

Quasi-static pressure-volume (PV) curves were generated from freshly extracted native lungs (from 300–400 g male rats), decellularized lungs, and day 7 engineered lungs prior to lobe freezing or fixation. The right lower lobe of native and decellularized lungs was tied off in order to match day 7 engineered lungs. Lungs were connected via trachea cannula to a syringe fitted with a pressure transducer (Edwards), with real-time monitoring in LabChart 8 (ADInstruments), then inflated three times with air to total lung capacity. To generate PV curves, lungs were then inflated stepwise to 1 mL total volume, followed by stepwise deflation to resting volume, with a 15 sec pause after each 0.1 mL volume change. Pressure measurements were captured from the end of each 15 sec interval, and plotted against lung volume. Three PV curves were generated for each lung. Quasi-static compliance was calculated from the slope of the opening limb of each PV curve, and is reported as the average of three technical replicates per lung.

### Bronchoalveolar lavage

Bronchoalveolar lavage (BAL) was performed on native lungs and day 7 engineered lungs after PV curve testing. One volume of PBS (equal to 0.7 × 30 mL/kg rat weight) was gently inflated and then suctioned via the trachea, collected on ice, and spun down to remove cells. BAL was analyzed by ELISA for SPB protein (LSBio) according to the manufacturer’s instructions.

### 3D lung alveolosphere assay

We modified the previously described alveolosphere assay^14^ to take advantage of the tri-culture, serum-free system described in this study. Tri-culture alveolosphere cultures were prepared in 0.4 μm, 24-well format cell culture inserts (Falcon) with 1:1 growth factor reduced Matrigel (Corning) to cell suspension in epithelium base medium (90 μL total volume, 2.25 × 10^4^ AEC2s, 2.25 × 10^4^ FBs, and 4.5 × 10^4^ RLMVECs per insert). Matrigel was allowed to solidify at 37 °C for 30 min, and then 0.5 mL medium was added to the outside of each insert. Culture medium was CK + DCIR or CK + DCIR with the specified medium component(s) removed (e.g. CK + DCIR without CHIR = “-CHIR”). Culture medium was changed on day 1 and every 2 days thereafter, and alveolospheres were analyzed after 7 days. 2–3 wells per condition were analyzed for each biological replicate, with reported quantification values (see details below) representing the average of technical replicate wells. Alveolospheres were imaged with an Axio Vert.A1 inverted microscope (Zeiss) and a Lumenera camera.

### Engineered lung (slice) tissue (ELT) culture

ELTs were prepared as previously described^75^. Briefly, native lungs were extracted from 300–400 g male rats as described for decellularized whole lung scaffold preparation, then inflated via the trachea with 2% LMP agarose in phenol red-free HBSS. The trachea was capped and the lung chilled on ice to allow the agarose to gel. The lobes were cut using a vibratome (Compresstome VF-300-0Z, Precisionary Instruments LLC) to 450 μm thick, and slices were snap frozen and stored at −80 °C until use (not more than 3 months).

Lung slices were thawed under PBS, then cut into 3-mm-wide strips and clipped into polytetrafluoroethylene (PTFE) tissue-culture cassettes^73^. Slices were decellularized in 6-well plates using a Triton X-100/SDC-based protocol adapted from our previously described whole lung decellularization protocol (see above and ref. ^36^). Decellularized slices were incubated with antibiotic/antimycotics for 48 h at 37 °C, then stored at 4 °C until seeding (not more than 30 days).

Tri-culture ELTs were prepared according to the same culture timeline and cell ratios used for tri-culture engineered whole lungs. Decellularized lung slices were rinsed 3 times with PBS, then transferred upside down to customized polydimethylsiloxane (PDMS) seeding baths^73^. Scaffolds were seeded on day −3 with ECs by pipetting a 100 μL cell suspension containing 5 M cells/mL of RLMVECs in endothelial medium (MCDB with 2% FBS, 1% P/S, and 0.1% gentamicin, 5 × 10^5^ cells per slice) directly onto the scaffold. After 2 h incubation at 37 °C, 900 μL fresh medium was added to each well. Endothelial medium was changed on day −2. On day 0, endothelial medium was removed, and the slices were seeded with a 100 μL cell suspension containing 2.5 M cells/mL AEC2s and 2.5 M cells/mL FBs in CK + DCIR medium (2.5 × 10^5^ AEC2s and 2.5 × 10^5^ AEC2s FBs per slice). The slices were incubated at 37 °C with 5% CO_2_ for 2 h, then 900 μL fresh CK + DCIR was added to each well. 24 h after seeding, the slices were removed from seeding baths, flipped, and transferred to well plates. The tissues were cultured in CK + DCIR medium until day 7, with medium changes every other day. On day 7, tissues were either saved for analysis or cultured for an additional 3 days (to day 10), either statically or under strain (see below), and in either CK + DCIR or DCIR medium (i.e. with CHIR and KGF removed).

ELTs were cultured under strain using an adaptation of our previously described bioreactor^74^. The bioreactor comprises a custom-made 3D-printed adapter (Formlabs Dental SG resin) which fits within a 6-well plate, allowing 6 ELTs to be stretched uniaxially in parallel. Each ELT is held by one fixed arm and one stainless-steel spring arm. The latter arm is driven by a voice coil linear actuator (BEI Kimco) controlled by a custom-programmed Pluto servo drive (Ingenia; programmed in MotionLab2). Tissues were cultured statically in CK + DCIR medium through day 7, then removed from their PTFE frames and loaded into the bioreactor for 3 additional days of culture under either 2.5% cyclic (4 cycles/min) or 2.5% tonic uniaxial strain.

For histological analysis and 5-ethynyl-2′-deoxyuridine (EdU) labeling, ELTs were incubated with 10 μM EdU for 2 h, then fixed with 10% NBF and paraffin embedded. For PCR analysis, RNA was extracted from fresh tissues and analyzed as described below; 2 ELTs were pooled per condition per experiment.

### Histology and immunofluorescent staining

Formalin-fixed tissues were routinely processed for paraffin embedding. Tissue sections were stained by standard methods for histological stains, or prepared for immunofluorescent (IF) staining. For IF, sections were baked for 45 min at 65 °C, deparaffinized and rehydrated, and subjected to heat-mediated antigen retrieval with citric acid. Sections were permeabilized with 0.2% Triton X-100 in PBS for 10–15 min, blocked with blocking buffer (5% BSA and 0.75% glycine in PBS) for 1 h at room temperature, and incubated with primary antibodies in blocking buffer overnight at 4 °C. Sections were rinsed and secondary antibodies applied for 1 h at room temperature, then stained with 4′,6-amidino-2-phenylindole (DAPI) and mounted with polyvinyl alcohol with DABCO (PVA-DABCO). A list of antibodies and concentrations used for IF can be found in Supplementary Table 1. For EdU staining, sections were processed according to the manufacturer’s instructions (Click-iT™ EdU Cell Proliferation Kit for Imaging, Alexa Fluor™ 647, Invitrogen). For TUNEL staining, sections were incubated with labeling solution following the above-described permeabilization step, according to the manufacturer’s instructions (Roche In Situ Cell Death Detection Kit, POD). Histological images were taken with an Axioskop 2 Plus upright microscope (Zeiss) and an AxioCam MRc camera (Zeiss), or with an EVOS FL Auto Imaging System (ThermoFisher Scientific) for whole lobe stitched images. Fluorescent images were taken with a Leica DMI6000 B inverted fluorescent microscope using a 40× oil objective (unless otherwise specified) and an Andor camera. Images were processed using ImageJ/Fiji. Any adjustments to brightness or contrast were applied equally across all experimental conditions in a given panel. In select instances, the contrast of individual color channels in a merged image was adjusted; this was performed identically across all experimental conditions and is noted in the corresponding figure legends.

### Oil Red O staining

Freshly isolated FBs were plated on a chamber slide (Lab-Tek II CC2, Nunc) and cultured for 72 h, then fixed in 4% paraformaldehyde for 20 min at room temperature. Cells were stained with 0.18% weight/volume Oil Red O (Sigma) in 60% isopropanol for 30 min, then counterstained with hematoxylin for 1 min.

### Quantitative real-time PCR

Cell samples, whole engineered lung, ELTs, and native rat tissue samples were homogenized in lysis Buffer RLT (Qiagen). Total RNA was isolated using the RNeasy Mini Kit (Qiagen) following the manufacturer’s instructions. RNA was reverse transcribed using the iScript cDNA Synthesis Kit (Bio-Rad), according to the manufacturer’s protocol. All PCR reactions were run in triplicate using 1 μL of cDNA in a 25 μL final volume with iQ SYBR Green Supermix (Bio-Rad) and 200 nM each of forward and reverse primers (see Supplementary Table 2). qRT-PCR was performed on the CFX96 Real-Time System (Bio-Rad) for 40 cycles. Average threshold cycle values (Ct) from triplicate PCR reactions were normalized to *Actb* (β-actin) for whole engineered lungs, or to *B2m* for ELTs (ΔCt) and reported as fold change using the 2^−ΔΔCt^ method. Gene expression in engineered whole lung samples was normalized to the relevant starting cell population (i.e. AEC2 isolate for epithelial markers; FB isolate for mesenchymal markers); native P7 and P60 rat lung gene expression is normalized to that of the average starting cell population and is presented for approximate comparison only. Gene expression in ELTs was normalized to corresponding day 7 control tissues from the same experiment; native rat lung gene expression is normalized to that of the average day 7 control tissue and is presented for approximate comparison only.

### Transmission electron microscopy

Tissue was fixed for 30 min at room temperature followed by 90 min at 4 °C in 2.5% glutaraldehyde and 2% paraformaldehyde in 0.1 M sodium cacodylate pH 7.4, and then rinsed 3 times in 0.1 M sodium cacodylate buffer. Processing and embedding of fixed tissue were performed by the Center for Cellular and Molecular Imaging, Electron Microscopy facility at Yale School of Medicine. Sections were imaged on a FEI Tecnai G2 Spirit BioTWIN Transmission Electron Microscope operated at 80 kV, using a Morada CCD camera (Olympus SIS).

### Sample preparation for scRNAseq

Freshly isolated AEC2s, freshly isolated FBs cultured for 72 h, or passage 5 RLMVECs were resuspended in 0.01% BSA in PBS, filtered through a 40 μm cell strainer, and diluted to 1 × 10^6^ cells/mL for single-cell library preparation.

Lungs from six postnatal day (P)7 Sprague-Dawley rat pups (3 males and 3 females) were dissociated for scRNAseq as previously described, with modifications^105^. Pups were given a dose of 400 U/kg IP heparin and euthanized via IP injection of sodium pentobarbital (150 mg/kg). Lungs were cleared of blood as described for the isolation of AEC2s, cannulated with a 24 G catheter (BD), and then inflated via the trachea with 2–3 mL of pre-warmed (37 °C) enzyme solution (1 mg/mL collagenase/dispase [Roche], 3 U/mL elastase [Worthington], 20 U/mL DNase [Worthington] in DMEM). The heart and large airways were dissected away and the remaining lung tissues and enzyme solution were collected and transferred to a shaking 37 °C water bath at 75 rpm for 20 min. The tissue was passed through a wire mesh strainer and the strainer was rinsed with ice-cold DMEM + 10% FBS + 1% P/S. The tissue solution was centrifuged for 5 min at 300 × *g*, resuspended in ACK lysing buffer (Lonza), and incubated for 90–120 s to lyse red blood cells. The cells were diluted in 0.01% BSA in PBS, spun down, and passed through a 70 μm filter. The cells were spun again for 3 min at 300 × *g*, resuspended in 0.01% BSA, then passed twice through 40 μm filters. The resulting single-cell suspension was counted, assessed for viability, then serially diluted to ≤1 × 10^6^ cells/mL for single-cell library preparation.

Approximately 1 cm^2^ from the right middle lobe of one AEC2/FB and one tri-culture engineered whole lung was dissociated for scRNAseq on day 7 of culture as described for P7 native rat lungs, with the following changes. Lung tissue was inflated with 5 mL of enzyme solution via repeated injection with a 25 G needle, then minced with fine scissors. The tissue and enzyme solution were collected, supplemented with an additional 5 mL of enzyme solution, and transferred to a shaking water bath as described above. No red blood cell lysis step was performed on engineered lung samples.

### Single-cell RNA sequencing and analysis

scRNAseq libraries were generated with the Chromium Single Cell 3′ v2 assay (for AEC2s and FBs), v3 assay (for native and engineered lung tissue), or v3.1 assay (for RLMVECs) according to the manufacturer’s protocol (10x Genomics). Samples were diluted for an expected cell recovery of 5,000 cells (AEC2s, FBs, and RLMVECs) or 10,000 cells (all other samples). Libraries were sequenced on an Illumina HiSeq 4000 (for AEC2s and FBs) or Illumina NovaSeq platform (for native and engineered lung tissue, and RLMVECs) with a targeted depth of 50,000 reads per cell. Alignment was performed using Cell Ranger 3.0.2 (10x Genomics), using Ensembl Rnor6.0 release 95.

Data was processed in R v3.6.1 using Seurat v3.1.1^106^. Cells were filtered to accept those having between 500–10,000 genes and between 500–100,000 UMI. Cells were additionally filtered based on the percentage of mitochondrial reads as follows: >1% and <7% for the AEC2 isolate; <5% for the FB isolate; <9% for RLMVECs; <28% for P7 native rat lung; <25% for d7 AEC2/FB engineered lung; and >6% and <28% for d7 tri-culture engineered lung. Expression matrices were normalized using the NormalizeData function and scaled with ScaleData, with regression on percentage mitochondrial reads, number of UMI counts, and cell cycle scoring. Each sample was clustered independently, and visualized using dimensionality reduction (UMAP, uniform manifold approximation and projection). As an additional filtration step, objects were subclustered by lineage (*Col3a1*^+^ mesenchymal cells [comprising fibroblasts, smooth muscle cells, pericytes, and mesothelium], *Epcam*^+^ epithelial cells, *Cdh5*^+^ endothelial cells, and *Ptprc*^+^ immune cells) to identify and remove suspected doublets or low-information cells. These filtered subsets were subsequently merged to create the full objects used for downstream analysis. Initial identities were designated by lineage assignment (see Supplementary Data 1 for cluster markers). Within the P7 native rat lung, the mesenchymal, epithelial, and endothelial lineages were subsetted and further clustered to assign subpopulation identities based on known markers (see Supplementary Data 1 for cluster markers).

Differential expression analysis was performed using FindAllMarkers in Seurat, using the default Wilcoxon Rank Sum test. Differentially expressed genes (DEGs) were defined as those genes expressed in a minimum of 10% of cells with fold change ≥1.5 and adjusted *P* < 0.05, unless otherwise specified. DEGs among AEC2/FB epithelium, tri-culture epithelium, and P7 AEC2s (Fig. 3e) were determined by performing each possible pairwise comparison, then removing duplicates to create a “consensus” gene list. Gene ontological and pathway enrichment was performed using Enrichr^107^. Heatmaps were created using the package ComplexHeatmap^108^. Lists of engineered lung cluster markers and epithelial and FB DEGs can be found in Supplementary Data 1–3. FB gene ontology and pathway enrichment data can be found in Supplementary Data 3.

Expression scoring for pre-defined gene sets was performed using the AddModuleScore function in Seurat (based on ref. ^109^). Gene sets were defined as follows: P7 endothelial subsets: top cluster markers among P7 endothelial populations, including shared “capillary” markers that are highly expressed in both general capillary ECs and aerocytes, compared to the other EC populations; P7 mesenchymal subsets: top cluster markers among P7 mesenchymal populations; unbiased FB activation score: ref. ^58^; TGFβ1/Smad3 targets: ref. ^51^; MANC signature: ref. ^27^. Gene sets are listed in Supplementary Data 5.

The R package connectome v0.1.9^61^ was used to map gene expression against rat homologs of literature-supported and putative ligand-receptor (LR) pairs within the FANTOM5 database^110^. Preliminary filtering of LR pairs was based on expression in a minimum of 10% of cells and adjusted *P* < 0.05 for both ligand and receptor (Supplementary Data 4). Differential expression of ligands and receptors was performed by applying FindAllMarkers for only those ligands and receptors expressed within AEC2/FB and tri-culture lungs (see Supplementary Data 4). Edge weights, for visualization purposes, were calculated as the sum of the normalized expression values for ligand and receptor for a given ligand/source cell-target cell/receptor edge.

### Image quantification

For alveolosphere number and diameter quantification, two 5× brightfield images were taken from each of 2–3 wells per condition after 7 days of culture, and alveolospheres of at least 50 μm in diameter were manually counted and measured in Fiji^111^. Each replicate data point represents the average from 2–3 wells per condition.

For quantification of total and proliferating AEC2s, or total FBs, in engineered whole lungs, 8 random 20× fields of view (FOV) were taken per sample (corresponding to 1954–14,738 cells/sample), avoiding the large airways and vessels, and cells were counted in Fiji. For quantification of EdU^+^ AEC2s in immunostained ELTs, 7–8 random 40x FOV were taken per sample (corresponding to 429–1622 cells/sample), and cells were counted manually in Fiji.

Image quantification was not performed blinded, as the same investigator both performed the experiments and analyzed the data, and phenotypic differences were in many cases obvious between conditions.

### Statistical analysis

Statistical analysis was performed in Prism v9.0.0 (GraphPad). In all cases, data points represent biological replicates, and statistical tests were performed only on biological replicates (*n* ≥ 3). No sample-size calculations were performed. Other than preliminary filtering of single-cell RNAseq data, no data were excluded. In instances in which technical replicates were averaged to derive the presented data points, this information is specified in the corresponding “Methods” section. The statistical tests used and exact value of *n* for each experiment are specified in the corresponding figure legend.

An unpaired two-tailed *t-*test was used for comparison of two groups, with the exception of analysis of scRNAseq cell scoring. A one-way analysis of variance (ANOVA) followed by Holm–Sidak’s multiple comparisons test for planned pairwise comparisons was used for comparisons of more than two groups with one factor, with the exception of the alveolosphere assay, which was analyzed according to a randomized block experimental design (with each independent experiment as a block), with a repeated measures ANOVA for comparison of multiple groups, followed by Holm-Sidak’s multiple comparisons test. Sphericity was assumed for randomized block experiments. For analysis of ELT experiments examining both stretch and medium factors, a two-way ANOVA with Holm–Sidak’s multiple comparisons test was used. For analysis of cell scoring using scRNAseq data, a Mann–Whitney test was used to compare two groups, and a Kruskal–Wallis test with Dunn’s post-tests was used to compare more than two groups.

For all tests, *P* < 0.05 was considered significant. For bar and scatter plots, center values represent the mean and error bars represent standard error of the mean (SEM). For boxplots, the center value represents the median; the limits of the box represent the 1st and 3rd quartile, respectively; and the whiskers extend to the minimum and maximum values. Outliers (defined as those data points greater than 1.5 times the interquartile range from the median) were removed from boxplots for visualization purposes only.

## Supplementary information

Supplementary Information

Supplementary Data 1

Supplementary Data 2

Supplementary Data 3

Supplementary Data 4

Supplementary Data 5

## Data Availability

The scRNAseq data generated in this study have been deposited in the Gene Expression Omnibus (GEO) database under accession code GSE178405. All data supporting the findings in this study are present within the article and associated supplementary information files. Additional data related to this paper and code necessary to reproduce this work are available from the corresponding author upon reasonable request.

## Code availability

All code used to analyze the data in this study is available from the corresponding author upon reasonable request.
