# Supplementary material for: Rational engineering of lung alveolar epithelium
Source: NPJ Regen Med. 2023 Apr 28;8:22. doi: 10.1038/s41536-023-00295-2 (PMC10147714; doi:10.1038/s41536-023-00295-2)
Supplement: Supplementary file 1 — Supplementary Information [file 41536_2023_295_MOESM1_ESM.pdf]

Supplementary Figure 1

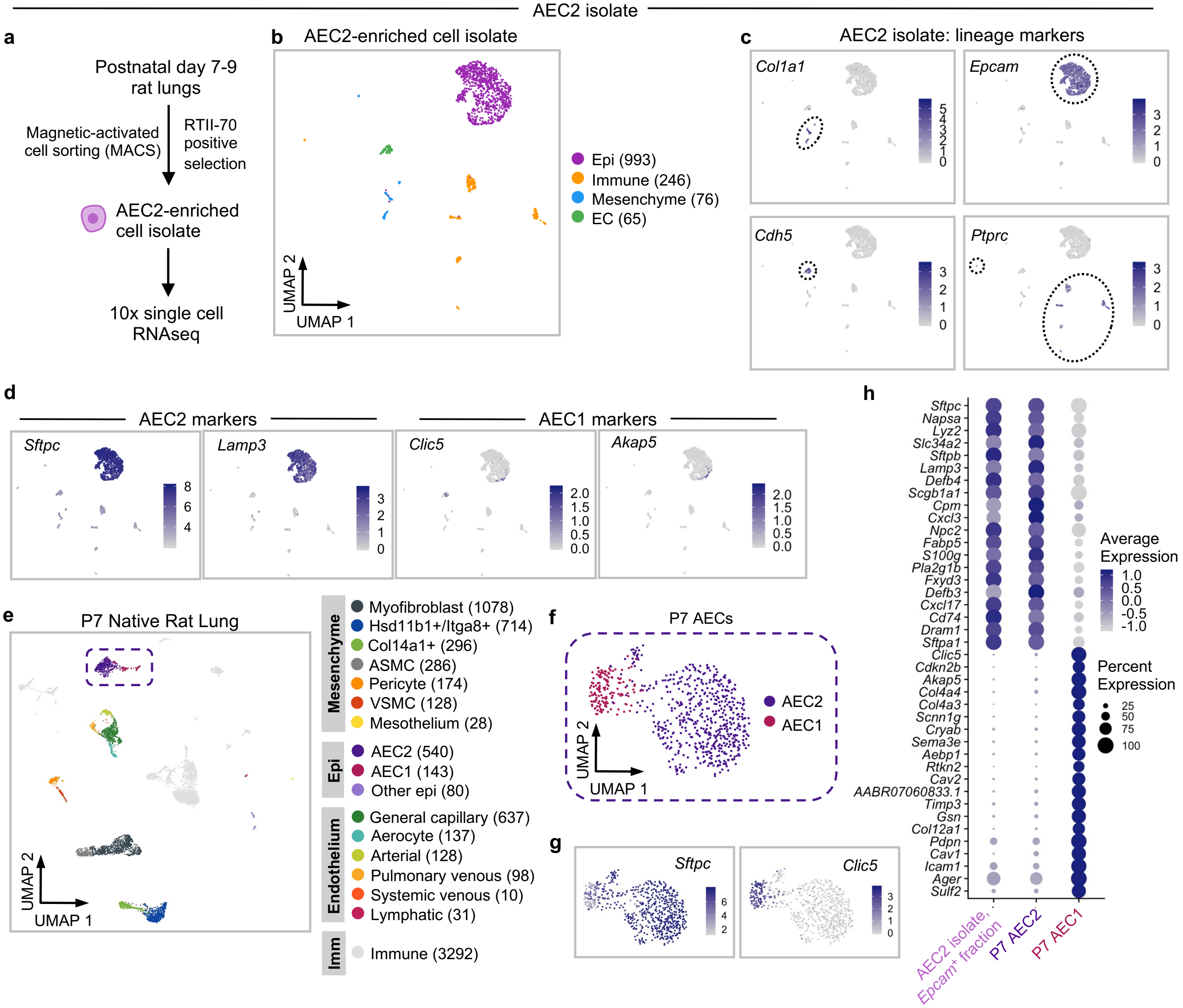

Supplementary Figure 1. Characterization of the AEC2 isolate. a Strategy for isolation of rat AEC2s. **b** UMAP embedding of scRNAseq data for freshly isolated AEC2s. **c, d** Expression data for example markers used to assign cluster labels (**c**) and specific AEC2 and AEC1 markers (**d**), projected onto the scRNAseq data from **b**. Dotted ovals in **c** encircle clusters positive for the indicated marker. **e** UMAP embedding of scRNAseq data for P7 whole rat lung dissociation. ASMC, airway smooth muscle cell. VSMC, vascular smooth muscle cell. **f** UMAP embedding of native P7 AEC2s and AEC1s, extracted from the full P7 data in **e** and re-clustered. **g** Expression data for specific AEC2 (*Sftpc*) and AEC1 (*Clic5*) markers projected onto the scRNAseq data from **f**. **h** Dot plot of gene expression for the top 20 P7 native AEC2 and AEC1 genes, demonstrating that the epithelial (*Epcam*$^{+}$) fraction of the AEC2 isolate comprises AEC2s. Numbers in parentheses in **b, e** indicate cell numbers per cluster after filtering. Epi, epithelium. EC, endothelial cell. Imm, Immune.

Supplementary Figure 2

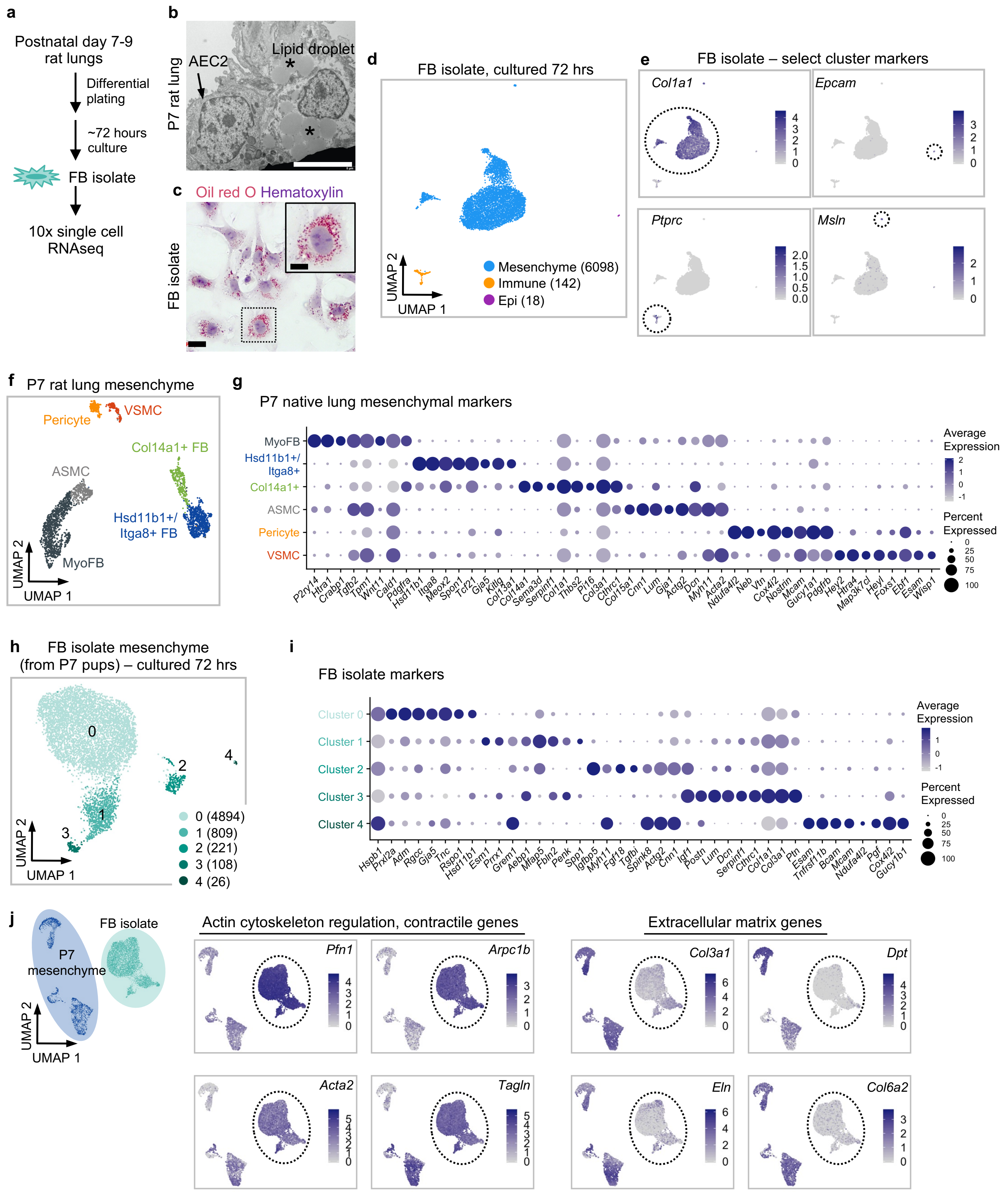

Supplementary Figure 2. Characterization of the fibroblast isolate. a Strategy for isolating lipofibroblast-enriched neonatal rat lung fibroblasts (FBs). Cells were expanded on tissue-culture plastic for 72 hours following isolation in order to achieve sufficient numbers for whole lung scaffold reseeding. **b** Transmission electron microscopy (TEM) of P7 rat lung demonstrating lipid-rich FBs neighboring an AEC2 at this timepoint.

Asterisk, lipid droplet. Scale bar, 5 µm. **c** Oil Red O staining of neutral lipid droplets in passage 0 FBs cultured for 72 hours. Scale bar, 20 µm; inset, 10 µm. **d** UMAP embedding of scRNAseq data for isolated FBs cultured for 72 hours. Epi, epithelium. **e** Expression data for select lineage markers (*Col1a1*, *Epcam*, *Ptprc*) and mesothelial marker *Msln* projected onto the scRNAseq data from **d**. *Msln*$^{+}$ mesothelial cells were excluded from downstream analyses of FB isolate mesenchymal clusters. Dotted ovals encircle clusters positive for the indicated marker. **f** UMAP embedding of native P7 lung mesenchyme (excluding mesothelium), extracted from the full P7 data in Supplementary Fig. 1e and re-clustered. ASMC, airway smooth muscle cell. MyoFB, myofibroblast. VSMC, vascular smooth muscle cell. **g** Dot plot showing scaled expression of select top markers from P7 mesenchyme clusters from **f**. **h** UMAP embedding of scRNAseq data for FB isolate mesenchymal cells (excluding mesothelial cells), extracted from the full FB isolate data in **d** and re-clustered. **i** Dot plot showing scaled expression of select top markers in FB isolate clusters from **h**. Note that following 72 hours of culture on tissue culture plastic, the FB isolate preserved features of native heterogeneity, including subpopulations sharing characteristic markers of Hsd11b1^{+}/Itga8^{+} FBs (Cluster 0), Col14a1^{+} FBs (Clusters 1 and 3), myofibroblasts and ASMCs (Cluster 2), and pericytes and VSMCs (Cluster 4). **j** Expression of select contractile (middle) and extracellular matrix genes (right), projected onto scRNAseq data of P7 mesenchyme and the FB isolate mesenchyme, demonstrating an overall shift toward a contractile and pro-migratory phenotype in the FB isolate. UMAP with cell identities is shown in left of panel; populations have been merged for purposes of expression level comparison. Dotted ovals encircle the FB isolate mesenchyme. Numbers in parentheses in **d, h** indicate cell numbers per cluster after filtering.

Supplementary Figure 3

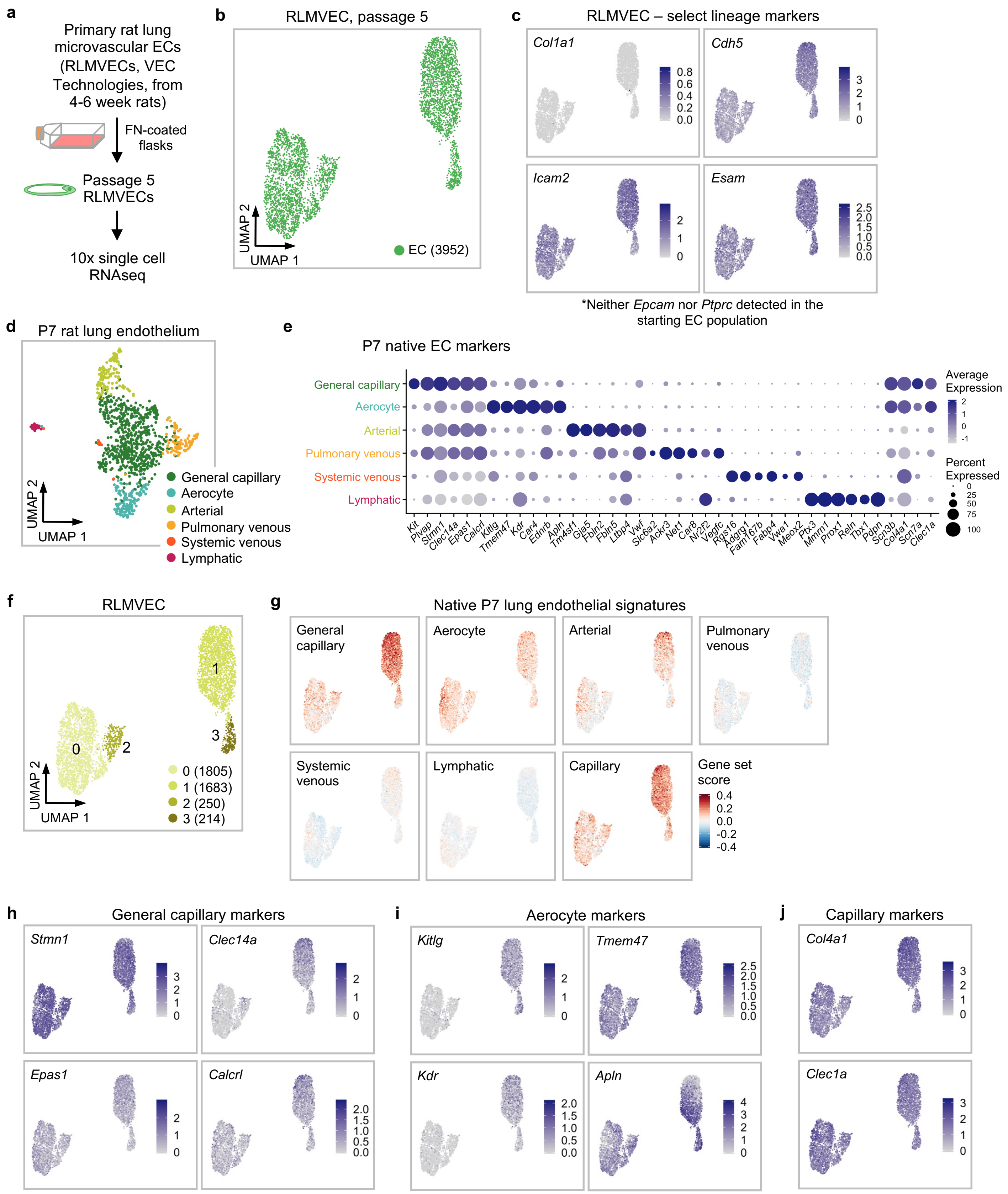

Supplementary Figure 3. Characterization of the starting rat lung microvascular endothelial cell population. a Details of primary endothelial cell (EC) population used for lung engineering. Rat lung microvascular endothelial cells (RLMVECs, VEC Technologies) were expanded on fibronectin (FN)-coated flasks to passage 5-6 prior to experimental use. **b** UMAP embedding of scRNAseq data for passage 5

RLMVECs, colored by cell lineage. **c** Expression data for mesenchymal marker *Col1a1* and endothelial lineage markers (*Cdh5*, *Icam2*, *Esam*) projected onto the RLMVEC scRNAseq data from **b**, demonstrating that RLMVECs are an endothelial population. Note that neither epithelial marker *Epcam* nor immune marker *Ptprc* was detected in the scRNAseq data. **d** UMAP embedding of native P7 lung endothelium, extracted from the full P7 data in Supplementary Fig. 1e and re-clustered. **e** Dot plot showing scaled expression of select top markers from P7 EC clusters from **d**, as well as select "capillary" markers (*Scn3b*, *Col4a1*, *Scn7a*, *Clec1a*) that are highly expressed in both general capillary ECs and aerocytes. **f** UMAP embedding of scRNAseq data for RLMVECs, subclustered from the data in **b**. **g** Scoring for P7 native lung EC subpopulation feature sets projected onto the RLMVEC scRNAseq data from **f**, demonstrating highest expression of general capillary and shared capillary features, with lower-level expression of aerocyte and arterial genes and minimal venous or lymphatic character in the starting endothelial population. Scores > 0 indicate enriched expression compared to random gene sets. **h, i, j** Expression data for select general capillary (**h**), aerocyte (**i**), and shared capillary (**j**) markers expressed by RLMVECs, projected onto the RLMVEC scRNAseq data from **f**. Numbers in parentheses in **b, f** indicate cell numbers per cluster after filtering.

Supplementary Figure 4

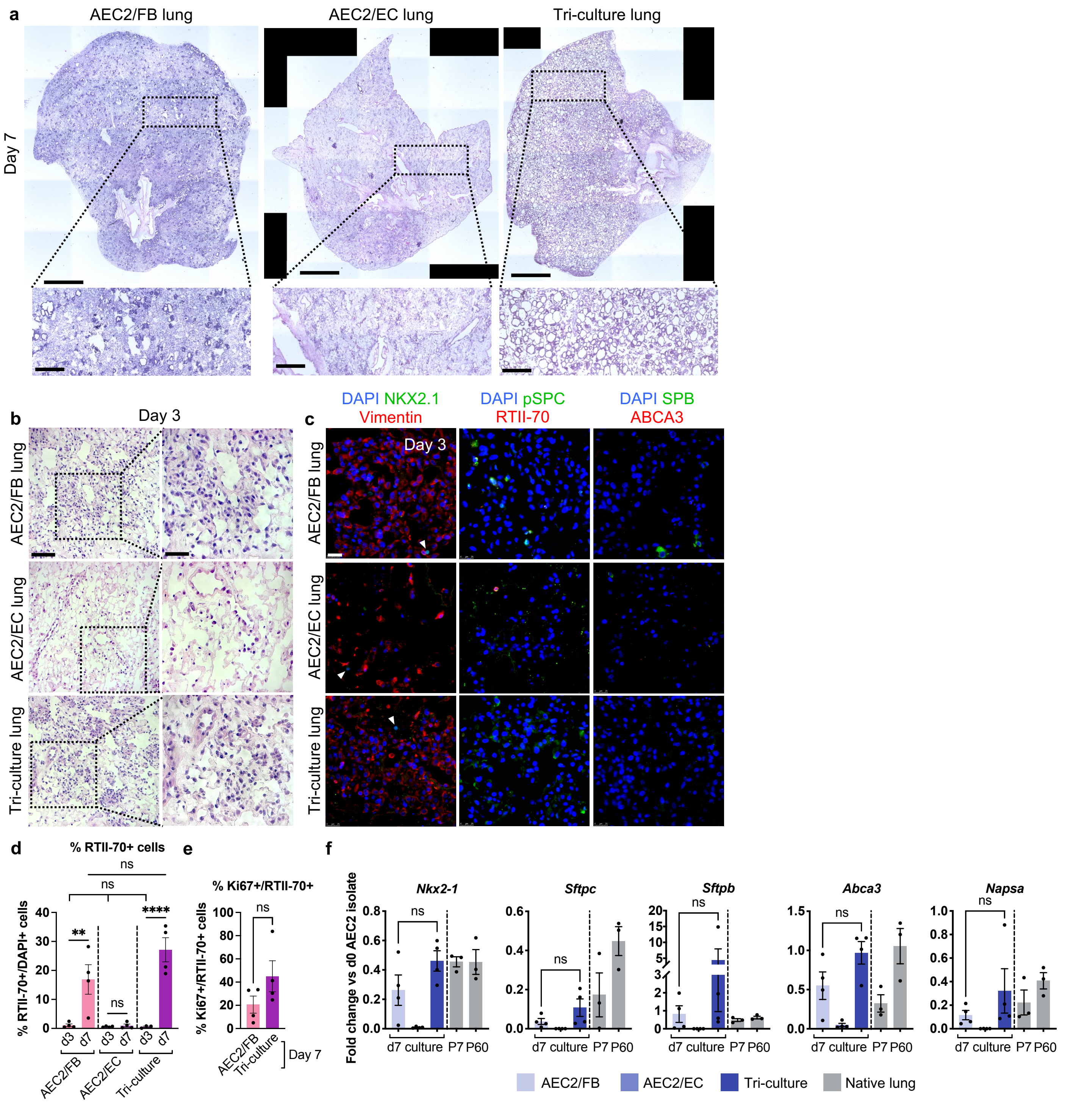

Supplementary Figure 4. Additional data on cellular repopulation of co- and tri-culture engineered lungs. **a** Whole lobe stitched H&E images of day 7 AEC2/FB, AEC2/EC, and tri-culture engineered lungs. Scale bars, 2 mm. Magnified region, 500 μm. **b** H&E staining of engineered lung cultures at day 3. Scale bars, 100 μm. Magnified region, 50 μm. **c** Immunostaining showing sparsity of AEC2s (by NKX2.1$^+$, pSPC$^+$/RTII-70$^+$, or SPB$^+$/ABCA3$^+$ staining) across all engineered lung conditions at day 3. **d, e** Quantification of total (**d**) and proliferating (**e**) RTII-70$^+$ cells in engineered lungs; *n* = 3 lungs: d3 tri-culture; *n* = 4: all other groups. **e** qRT-PCR of AEC2 gene expression in day 7 engineered lungs; *n* = 4 lungs. Gene expression in native P7 and P60 rat lungs is normalized to the average expression in day 0 AEC2 isolates and shown for approximate comparison only; *n* = 3 lungs. Error bars indicate the mean ± SEM. **d** One-way ANOVA with Holm-Sidak's multiple comparisons test. **e, f** Unpaired two-tailed *t*-test. ns, not significant, $^{**}P < 0.01$, $^{****}P < 0.0001$.

Supplementary Figure 5

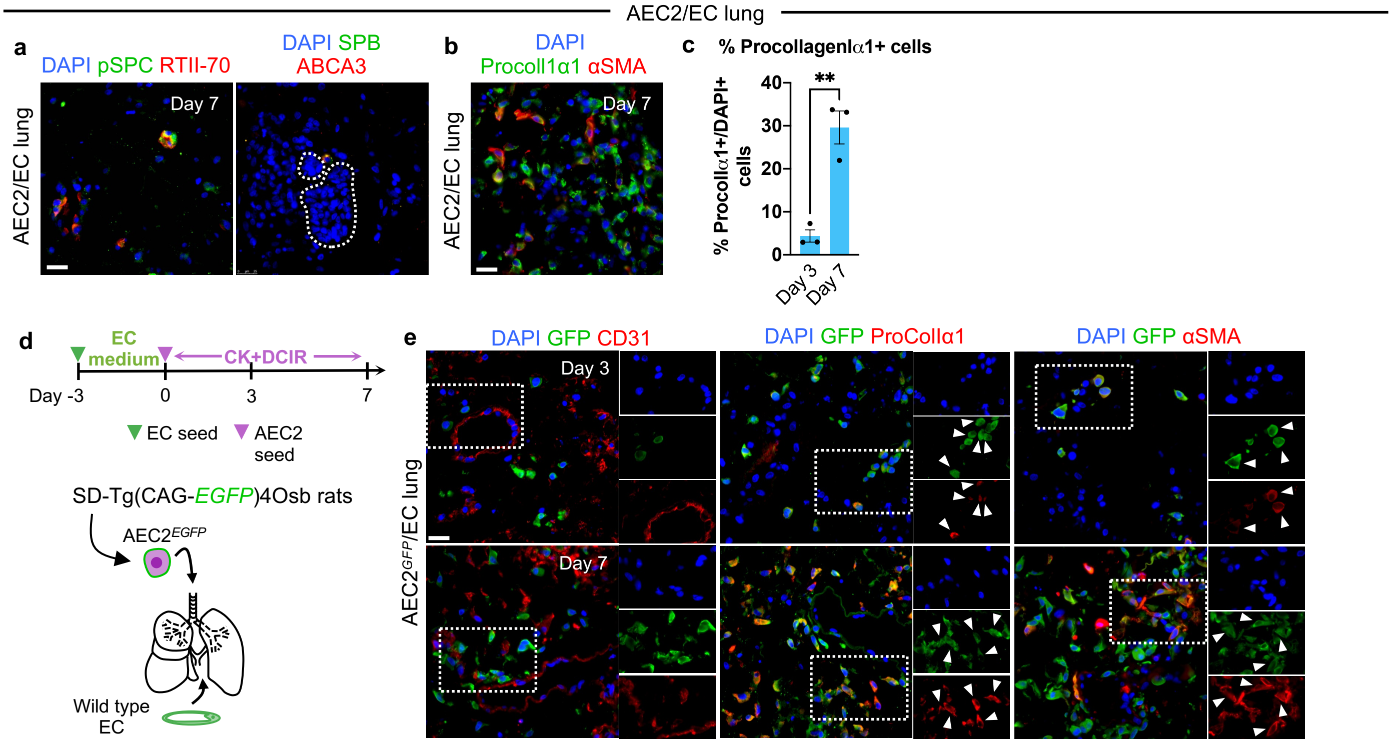

Supplementary Figure 5. Fibroblast-like cells in AEC2/EC lungs are derived from the AEC2 isolate. a Immunofluorescent staining for AEC2s (pSPC^{+}/RTII-70^{+} or SPB^{+}/ABCA3^{+}) in day 7 AEC2/EC lungs. Dotted lines outline cell clusters with epithelial morphology that fail to express AEC2 markers. **b** Immunofluorescent staining for procollagen Iα1 (ProCollα1) and alpha smooth muscle actin (αSMA) in day 7 AEC2/EC lungs. **c** Quantification of ProCollα1^{+} cell numbers in AEC2/EC engineered lungs, showing an increase in the number of FB-like cells by day 7 of culture; $n = 3$ biological replicates. Error bars indicate the mean ± SEM. Unpaired two-tailed t-test. $^{**}P < 0.01$. **d** Timeline for AEC2^{GFP}/EC lung culture, in which AEC2s isolated from *EGFP*$^{+}$ rats were cultured together with wild-type ECs; $n = 1$. All GFP^{+} cells in the lung culture will have arisen from the AEC2 isolate (which does include contaminating non-AEC2 cells; see Supplementary Fig. 1b, c). **e** Immunofluorescent staining of day 3 and 7 AEC2^{GFP}/EC lungs showing GFP^{+} staining co-localizing with FB markers (ProCollα1 and αSMA – see arrowheads) but not with CD31, indicating that the FB-like cells arose from cells of the GFP^{+} isolate. Scale bars, 25 µm.

Supplementary Figure 6

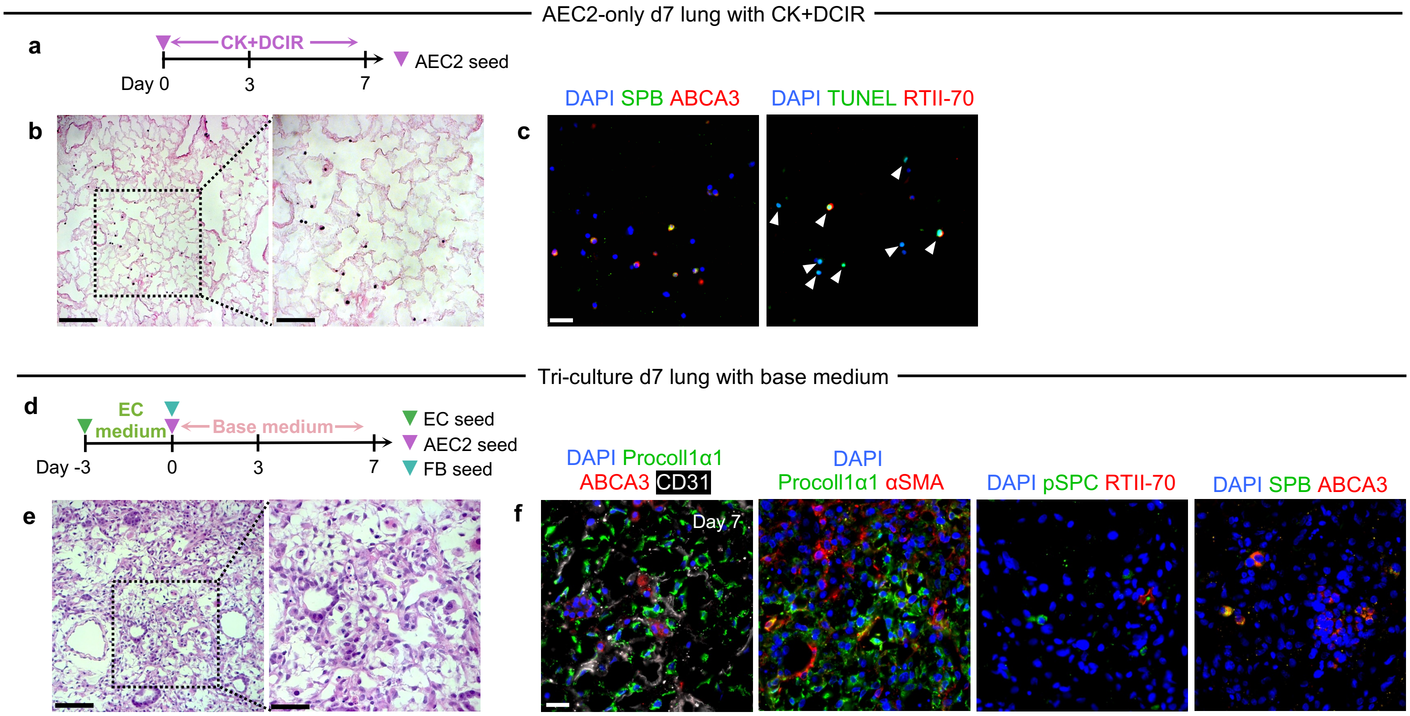

Supplementary Figure 6. CK+DCIR medium and endogenous tri-culture signaling in base medium are insufficient for organized scaffold repopulation with AEC2s. a Timeline for AEC2-only (monoculture) engineered lung with CK+DCIR medium; $n = 2$ biological replicates. **b** H&E staining shows sparse cellularity after 7 days of AEC2 monoculture. Scale bars: main image, 100 µm. Magnified region, 50 µm. **c** Immunofluorescent staining showing that SPB^{+}/ABCA3^{+} and RTII-70^{+} AEC2s are present in day 7 AEC2-only monoculture, but that many are apoptotic by terminal deoxynucleotidyl transferase dUTP nick end labeling (TUNEL) staining. Arrowheads, TUNEL^{+} nuclei. Scale bar, 25 µm. **d** Timeline for tri-culture engineered lung cultured without CK+DCIR medium additives (*i.e.* with CK+DCIR base medium only after day 0); $n = 1$. **e** H&E staining of tri-culture lung cultured with base medium only reveals few cuboidal epithelial-like cells at 7 days. Scale bars: main image, 100 µm. Magnified region, 50 µm. **f** Immunostaining of tri-culture lung with base medium shows relatively few scattered pSPC^{+}, RTII-70^{+}, or SPB/ABCA3^{+} AEC2s within a disorganized stroma of CD31^{+} ECs and Procoll1α1^{+} and/or αSMA^{+} FBs. Scale bar, 25 µm.

Supplementary Figure 7

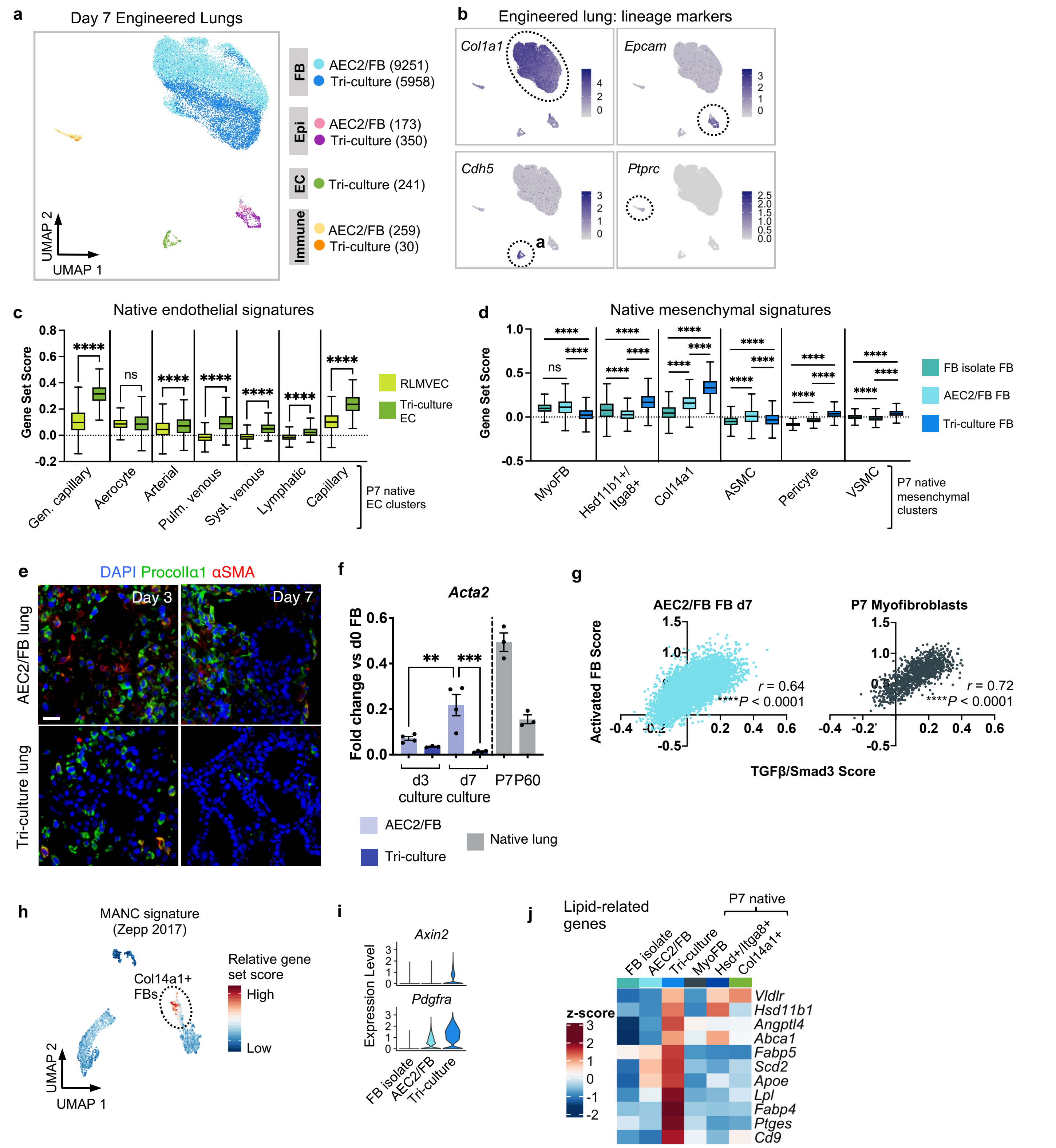

Supplementary Figure 7. Additional engineered, native, and cell isolate scRNAseq data. a UMAP embedding of scRNAseq data for AEC2/FB and tri-culture day 7 engineered lungs. Samples were clustered independently but integrated here for aid of visualization. Numbers in parentheses indicate cell numbers per cluster after filtering. **b** Expression data for example markers used to assign cells to fibroblast (FB), epithelial (epi), endothelial cell (EC), or immune lineages in engineered lungs, projected onto scRNAseq data from **a**. Dotted ovals encircle clusters positive for the indicated marker. Note that the scRNAseq data revealed a cluster of *Ptprc*$^+$ immune cells in each lung, most likely derived from the AEC2 cell isolate (see Supplementary Fig.

1b, c). While we cannot exclude a role for these immune cells in the observed lung phenotypes, they were not included in the analyses in this study. **c** Scoring of individual starting RLMVECs and tri-culture lung ECs for expression of P7 native endothelial cluster gene sets (see Supplementary Fig. 3d). Mann-Whitney test. **d** Scoring of individual FBs for expression of P7 native mesenchymal cluster gene sets (see Supplementary Fig. 2f). MyoFB, myofibroblast. ASMC, airway smooth muscle cell. VSMC, vascular smooth muscle cell. Kruskal-Wallis test with Dunn's post-test. In **c, d** Scores > 0 indicate enriched expression compared to random gene sets. For boxplots, the center value represents the median; the limits of the box represent the 1st and 3rd quartiles; and the whiskers extend to the minimum and maximum values (outliers not shown). **e** Immunostaining of day 3 and day 7 engineered lungs for procollagen Iα1 (ProcolIα1) and αSMA (gene *Acta2*). Scale bar, 25 µm. **f** qRT-PCR analysis for *Acta2* in engineered lungs, showing that *Acta2* expression in co-culture lungs increased over time and was significantly greater than that in tri-culture lungs at day 7; $n = 3$ biological replicates, d3 tri-culture, P7 and P60 native; $n = 4$, all other conditions. Native gene expression is normalized to the average expression in day 0 FB isolates and shown for approximate comparison only. Error bars indicate the mean ± SEM. One-way ANOVA with Holm-Sidak's multiple comparisons test. **g** Scatterplots with associated Spearman correlation coefficients r showing a moderate correlation between TGFβ/Smad3 target expression and activation scores in AEC2/FB FBs, similar to that observed in P7 native lung myofibroblasts. **h** Scoring for the mesenchymal alveolar niche cell (MANC) feature set projected onto P7 mesenchyme scRNAseq data (see UMAP in Supplementary Fig. 2f and UMAP inset in Fig. 4a), demonstrating that the Col14a1$^+$ FBs in our native lung dataset are most similar to MANCs. **i** Violin plots of expression of MANC signature genes *Axin2* and *Pdgfra* in the starting FB isolate and engineered lung FBs. **j** Heatmap of lipid-related genes enriched in tri-culture FBs. ns, not significant; $^{**}P < 0.01$, $^{***}P < 0.001$, $^{****}P < 0.0001$.

Supplementary Figure 8

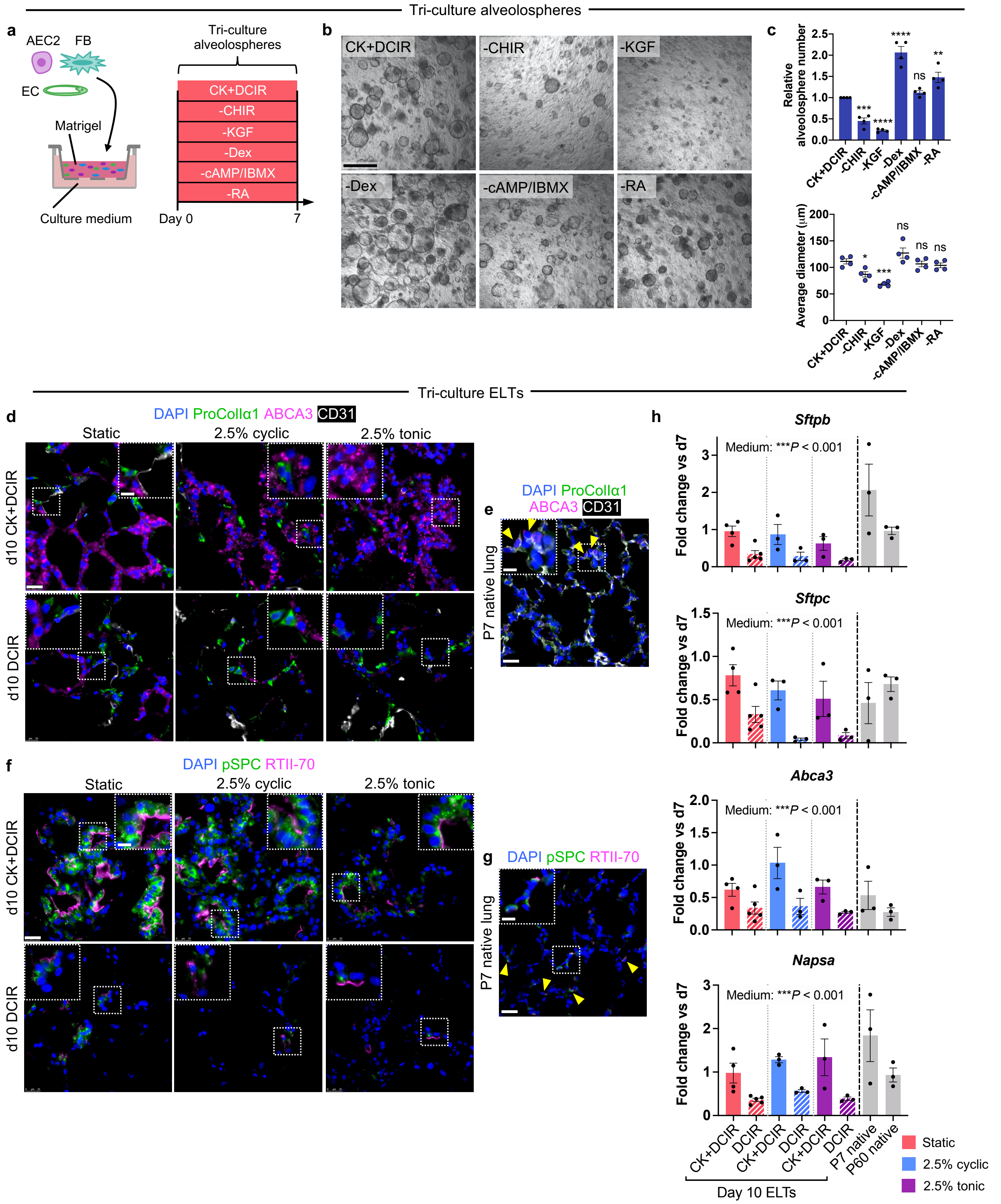

Supplementary Figure 8. CHIR and KGF withdrawal is associated with a loss of AEC2 phenotype in tri-culture ELTs, regardless of stretch. a Schematic of 3D alveolosphere assay to test the contribution of individual culture medium supplements to AEC2 growth in the tri-culture setting. The same cell populations

used for whole lung engineered cultures were suspended in Matrigel and cultured for 7 days in complete CK+DCIR medium, or with CK+DCIR with the specified medium component(s) excluded. CHIR, CHIR99021. KGF, keratinocyte growth factor. Dex, dexamethasone. cAMP, cyclic AMP. IBMX, 3-isobutyl-1-methylxanthine. RA, retinoic acid. **b, c** Phase contrast images (**b**) and quantification (**c**) of day 7 alveolospheres from assay in (**a**); $n = 4$. **c** Repeated measures one-way ANOVA with Holm-Sidak's multiple comparisons test. Error bars: mean ± SEM. P values given for comparison versus CK+DCIR control. ns, not significant, $^{*}P < 0.05$, $^{**}P < 0.01$, $^{***}P < 0.001$, $^{****}P < 0.0001$. Scale bars, 500 μm. **d, e** Immunostaining for AEC2s (ABCA3), FBs (ProColIα1), and ECs (CD31) in tri-culture d10 engineered lung tissues (ELTs) (**d**) or P7 native lung (**e**)**. f, g** Immunostaining for AEC2-specific markers pSPC and RTII-70 in tri-culture d10 ELTs (**f**) or P7 native lung (**g**). **d-g** Scale bars: main image, 25 μm; magnified region, 10 μm. Arrowheads, AEC2s localized to alveolar corners. Note that imaging parameters used for the native tissues in **e** and **g** differ from those used in the corresponding ELTs, as higher exposures were generally required for native lung samples to visualize the proteins. **h** qRT-PCR of AEC2 gene expression in d10 ELTs, normalized to their respective d7 control tissues. Data for static controls are repeated from Fig. 6f. Native gene expression is normalized to the average expression in d7 tissues and shown for approximate comparison only. Static/CK+DCIR: $n = 4$; static/DCIR: $n = 5$; all other conditions: $n = 3$. Error bars: mean ± SEM. Text annotation indicates significance by two-way ANOVA for medium main effect ("medium").

Supplementary Table 1. Antibodies and dilutions for immunofluorescent staining

Primary Antibody	Host species	Supplier	Product number	Dilution	Clone
Anti-αSMA	Mouse	Dako	M0851	1:2000	1A4
Anti-ABCA3	Mouse	Abcam	ab24751	1:50	3C9
Anti-AGER	Goat	R&D Systems	AF1145	1:200	N/A
Anti-AQP5	Rabbit	Millipore	AB3559	1:500	N/A
Anti-CD31	Goat	R&D Systems	AF3628	5 µg/mL	N/A
Anti-GFP	Mouse	Santa Cruz	sc-9996	1:50	B-2
Anti-GFP	Rabbit	Abcam	ab290	1:5000	N/A
Anti-Laminin	Rabbit	Abcam	ab11575	1:200	N/A
Anti-Laminin beta 2	Mouse	LSBio	LS-C25105	1:200	2Q592
Anti-NKX2.1	Rabbit	Abcam	ab76013	1:100	EP1584Y
Anti-procollagenIα1	Rabbit	Rockland	600-401-D19	1:50	N/A
Anti-pSPC	Rabbit	Millipore	AB3786	1:1000	N/A
Anti-RTI-40	Mouse	Terrace Biotech	TB-11ART1-40	1:200	N/A
Anti-RTII-70	Mouse	Terrace Biotech	TB-44ART2-70	1:40	N/A
Anti-SPB	Rabbit	Santa Cruz	sc-13978	1:50	H-300
Anti-TGFβ1	Rabbit	Abcam	ab92486	1:200	N/A
Anti-Vimentin	Mouse	Abcam	ab8069	1:200	V9
Secondary Antibody					
Anti-goat IgG, Alexa Fluor 488	Chicken	Invitrogen	A21467	1:500	N/A
Anti-goat IgG, Alexa Fluor 555	Donkey	Invitrogen	A21432	1:500	N/A
Anti-goat IgG, Alexa Fluor 647	Donkey	Invitrogen	A-21447	1:500	N/A
Anti-mouse IgG, Alexa Fluor 555	Goat	Invitrogen	A21424	1:500	N/A
Anti-mouse IgG, Alexa Fluor 568	Donkey	Invitrogen	A10037	1:500	N/A
Anti-mouse IgG, Alexa Fluor 647	Goat	Invitrogen	A-21235	1:500	N/A
Anti-rabbit IgG, Alexa Fluor 488	Chicken	Invitrogen	A21441	1:500	N/A
Anti-rabbit IgG, Alexa Fluor 488	Goat	Invitrogen	A11034	1:500	N/A
Anti-rabbit IgG, Alexa Fluor 555	Goat	Invitrogen	A21429	1:500	N/A
Anti-rabbit IgG Alexa Fluor 647	Donkey	Invitrogen	A31573	1:500	N/A

Supplementary Table 2. qRT-PCR primer sequences

Rat Primer	Forward	Reverse
Abca3	GAGGTCTTCCTTCGGGTTGG	GTCCATCACCCCACACAAGT
Acta2	GCTTTGCTGGTGATGATGCT	GATCCCTCTCTTGCTCTGC
Actb	GCAGGAGTACGATGAGTCCG	ACGCAGCTCAGTAACAGTCC
Ager	AGAAACCGGTGATGAAGGACA	GGTTGTCGTTTTCGCCACAG
B2m	CCGTGATCTTTCTGGTGCTT	ATTTGAGGTGGGTGGAACTG
Clic5	CTGGCCGACTGCAATCTACT	GTGAACTCGTCCCGTGCATA
Napsa	CAGGTCCACATGCAGAGTGT	GCCTTATTCAAGGCCCGGAT
Nxk2-1	TGCTTTATGGTCGGACCTGG	TTGCGGAGGGTAGAGGGAAA
Pdpn	AGTGTTGCTCTGGGTTTTGG	GGGTTCACCATGTCATCTCC
Sftpb	CCTGGCTGAGCGTTACACA	TTCAATCAGAGGCTCCAGAG
Sftpc	CTCCTGACCGCCTATAAGC	TGGCCTGGAAGTTCTTGAAT

List of Supplementary Data files (separate Excel spreadsheets):

Supplementary Data 1. scRNAseq cluster markers for engineered and P7 native lung
Single-cell RNAseq cluster markers for day 7 AEC2/FB engineered lung (tab 1), day 7 tri-culture engineered lung (tab 2) and P7 native lung (tab 3)

Supplementary Data 2. Consensus DEGs for engineered and native epithelium
Key to heatmap clusters (corresponds to Fig. 3e; tab 1) and differentially expressed genes (DEGs) by heatmap cluster (tab 2)

Supplementary Data 3. scRNAseq fibroblast analysis
Single-cell RNAseq cluster markers for FB isolate, engineered FBs, and P7 native FBs (corresponds to clusters in Fig. 4a; tab 1); differentially expressed genes (DEGs; tab 2) with associated Hallmark pathway enrichment (tab 3) enriched in the FB isolate and day 7 AEC2/FB FBs, compared to day 7 tri-culture FBs (corresponds to Fig. 4c); and biological process (BP) enrichment in day 7 AEC2/FB FBs (tab 4) and day 7 tri-culture FBs (tab 5; corresponds to Fig. 4d)

Supplementary Data 4. Engineered and native lung connectomic data
Connectomes for day 7 AEC2/FB engineered lung (tab 1), day 7 tri-culture engineered lung (tab 2), and P7 native lung (tab 3); and differentially expressed ligands and receptors (LR) among epithelium (AEC2/FB vs tri-culture, tab 4) and fibroblasts (AEC2/FB vs tri-culture, tab 5)

Supplementary Data 5. Gene sets used for cell scoring
